# Antioxidant and Anti-inflammatory Properties of Resveratrol in Diabetic Nephropathy: A Systematic Review and Meta-analysis of Animal Studies

**DOI:** 10.3389/fphar.2022.841818

**Published:** 2022-03-09

**Authors:** Heng-Chang Hu, Yuan-Hong Lei, Wei-Hua Zhang, Xiao-Qiong Luo

**Affiliations:** ^1^ College of Basic Medicine, Chengdu University of Traditional Chinese Medicine, Chengdu, China; ^2^ Department of Neurology, Chongqing Traditional Chinese Medicine Hospital, Chongqing, China

**Keywords:** resveratrol, diabetic nephropathy, plant-derived agents, anti-inflammatory, antioxidant, meta-analysis

## Abstract

**Background:** Accumulated experimental evidence suggests that resveratrol may have an effect on diabetic nephropathy by inhibiting inflammation and decreasing oxidative stress. However, the credibility of the evidence for this practice is unclear. Thus, we aimed to perform a systematic review and meta-analysis of animal studies to evaluate the antioxidant and anti-inflammatory properties of resveratrol when used in the treatment of diabetic nephropathy.

**Methods:** Electronic bibliographic databases including PubMed, EMBASE, and Web of Science were searched for relevant studies. The methodological quality of animal studies was assessed based on the SYstematic Review Center for Laboratory animal Experimentation Risk of Bias (SYRCLE’s RoB) tool. A meta-analysis was performed based on the Cochrane Handbook for Systematic Reviews of Interventions by using RevMan 5.4 software. This study was registered within International Prospective Register of Systematic Reviews (PROSPERO) as number CRD42021293784.

**Results:** Thirty-six qualified studies involving 726 animals were included. There was a significant association of resveratrol with the levels of blood glucose (BG), serum creatinine (Scr), blood urea nitrogen (BUN), catalase (CAT), superoxide dismutase (SOD), malondialdehyde (MDA), glutathione (GSH), glutathione peroxidase (GPx), and interleukin-1β (IL-1β). Nevertheless, resveratrol treatment did not effectively decrease the levels of tumor necrosis factor-α (TNF-α) and interleukin-6 (IL-6). In addition, more remarkable antioxidant and hypoglycemic effects were observed in type 2 diabetic nephropathy rather than in type 1 diabetic nephropathy based on subgroup analysis.

**Conclusion:** In this meta-analysis, resveratrol can exert its antioxidant activities by reducing the levels of MDA and recovering the activities of SOD, CAT, GSH, and GPx. With regard to pro-inflammatory cytokines, resveratrol had a positive effect on the reduction of IL-1β. However, the analysis indicated that resveratrol had no effect on IL-6 and TNF-α levels, probably because of the methodological quality of the studies and their heterogeneity. Current evidence supports the antioxidant and anti-inflammatory properties of resveratrol, but its relationship with the levels of some inflammatory cytokines such as IL-6 and TNF-α in animals with diabetic nephropathy needs further elucidation.

## Introduction

Diabetic nephropathy (DN), one of the most common, serious, and expensive diabetic complications (Alicic et al., 2021), is a chronic progressive disorder that can result in kidney failure and is currently the primary cause of kidney replacement therapy worldwide (DeFronzo et al., 2021). Multiple factors including hyperglycemia, inflammation, and oxidative stress contribute to the pathogenesis and progression of DN (Singh et al., 2011; Markus et al., 2018; DeFronzo et al., 2021). Literature data show that oxidative stress and inflammation could directly damage renal function in streptozotocin (STZ)-induced diabetic animals leading to DN (Barman et al., 2018). Current treatments for DN, including optimized glycemic control and blood pressure control with renin-angiotensin system blockade (angiotensin-receptor blockers or angiotensin-converting enzyme inhibitors), are insufficient to delay the progression to end-stage renal disease (ESRD) in a substantial proportion of patients (Fernandez-Fernandez et al., 2014; Lytvyn et al., 2020). Therefore, it is imperative to establish novel treatment approaches for the management of DN.

To achieve this, Chinese herbal medicine (CHM) and its bioactive ingredients as natural treatment options have become the focus of DN drug research. In comparison with conventional drugs, they typically do not selectively inhibit one molecular target but exhibit a pleiotropic action profile (Meresman et al., 2021), which may simultaneously affect fundamental processes in the pathogenesis of DN and achieve higher efficacy in the treatment of DN. *Polygonum cuspidatum*, the dry rhizome and root of *Polygonum cuspidatum* Sieb. et Zucc., is well known as Hu Zhang in China and widely used as CHM to treat diseases such as jaundice, fever, and cough (Peng et al., 2013). *Polygonum cuspidatum* contains a variety of bioactive ingredients such as polydatin and resveratrol (RSV), and is the primary source of the extraction of RSV (Zhang et al., 2020). Modern pharmacological studies suggested that RSV has a beneficial influence on DN with its powerful anti-inflammatory and antioxidant properties. In animal models of DN, RSV treatment can significantly relieve kidney damage by increasing the activity of antioxidant enzymes, such as superoxide dismutase (SOD), catalase (CAT), and glutathione peroxidase (GPx) (Khamneh et al., 2012; Ramar et al., 2012). Moreover, it was suggested that RSV has renal protective effects by reducing the levels of malondialdehyde (MDA) (Zhang et al., 2019; Wang et al., 2020). In addition, RSV can improve STZ-mediated DN symptoms in animals through decreasing the inflammatory cytokines, such as tumor necrosis factor-α (TNF-α), interleukin-1β (IL-1β), and interleukin-6 (IL-6) (Koca et al., 2016; Ma et al., 2016). Results of these animal experiments demonstrated a protective role of RSV in DN and suggested that the antioxidant and anti-inflammatory properties were implicated in the underlying mechanisms. However, these findings in the individual animal experiments are often affected by multiple factors, such as small sample sizes, DN animal models, intervention duration, and small-study effects, so it is insufficient to draw reliable conclusions about the antioxidant or anti-inflammatory properties of RSV in the treatment of DN according to this low-quality evidence. In addition, dose-response effects and time-response effects play a critical role in the treatment of DN, but it is difficult to determine the appropriate dosage and intervention duration of RSV based on the individual animal experiments. Moreover, whether RSV has adverse effects in the management of DN remains uncertain. Furthermore, methodological quality of the animal experiments and publication bias are still unclear, which may exaggerate the RSV efficacy.

Reviews based on preclinical animal data can address these aforementioned problems, assist in determining what is valuable in further research, and inform future clinical trials. In order to bridge the gap between the animal studies and clinical application, we performed a systematic review and meta-analysis of preclinical animal data to evaluate RSV in the management of DN. The purposes of this study were to 1) provide empirical evidence to assess the antioxidant and anti-inflammatory activities of RSV in the treatment of DN, 2) discuss the appropriate dosage and intervention duration of RSV on DN, 3) provide information on adverse effects of RSV in the treatment of DN, 4) provide an assessment of methodological quality of the animal studies and publication bias, and 5) provide suggestions for future animal studies and clinical trials.

## Materials and Methods

This systematic review and meta-analysis was performed according to the Cochrane Handbook for Systematic Reviews of Interventions and reported based on Preferred Reporting Items for Systematic Reviews and Meta-analyses guidelines (Page et al., 2021). The protocol for this systematic review and meta-analysis is available on the website of PROSPERO (CRD42021293784).

### Search Strategies

Electronic bibliographic databases including PubMed, EMBASE, and Web of Science were searched for relevant studies published from January 2010 to September 2021. The language was limited to English. Medical subject headings (MeSH) and free words for database searches were as follows: Subjects (diabetic nephropathies (MeSH), “diabetic renal disease,” “kidney diseases, diabetic,” “kidney disease, diabetic,” “diabetic kidney diseases,” “diabetic kidney disease,” “nephropathies, diabetic,” “diabetic nephropathy,” “nephropathy, diabetic,” “DKD,” “DN; ” Intervention [resveratrol (MeSH), “trans resveratrol,” “cis resveratrol”].

### Inclusion Criteria

1) Subjects: all animal models with DN; 2) Intervention: RSV with all dosage and duration; 3) Control: same solvent (e.g., water and saline), no intervention, etc.; 4) Outcomes: blood glucose (BG), serum creatinine (Scr), and blood urea nitrogen (BUN) were the primary outcomes, IL-1β, IL-6, TNF-α, SOD, MDA, CAT, GSH, and GPx were the secondary outcomes; 5) Study design: controlled studies with a separate control group; 6) Language: English.

### Exclusion Criteria

1) Subjects: animals with co-morbidity, clinical trials, and *in vitro* models; 2) Intervention: RSV without batch number; 3) Control: other preparation of RSV (some pharmaceutical preparation or nutritional products containing RSV); 4) Study design: case studies, cross over studies, and studies without a separate control group; 5) Not an original full research paper (e.g., review, editorials/letters, abstracts); 6) Duplicate publication; 7) Studies without full text.

### Study Selection and Data Extraction

The screening of studies was performed in two phases, initial screening based on title and abstract, followed by full-text screening of the potentially eligible articles for final determination (Higgins and Green, 2021). In each phase, two reviewers independently assessed each study. Disagreements about whether a study should be included were resolved through discussion with a third reviewer.

Two reviewers extracted the following data independently from included studies: 1) Basic information: first author’s surname and year of publication; 2) Information on subjects: sample size, weight, species, and animal models of DN in the experimental group and control group; 3) Information on treatment: intervention duration and dose; 4) Outcome measures: BG, Scr, BUN, TNF-α, IL-6, IL-1β, CAT, GSH, SOD, MDA, and GPx. All the outcome measures were continuous data, so the mean and the standard deviation for each intervention group were extracted. For studies with multiple experimental groups paired with one control group, then this control group was divided up approximately evenly among the comparisons and each pair-wise comparison was involved in the meta-analysis (Higgins and Green, 2021). In case outcomes were presented at multiple time points, the data were extracted from the last time point. Any disagreements between reviewers over the data extraction were resolved through discussion with a third reviewer.

### Quality Assessment

The methodological quality of the included studies was assessed according to the SYstematic Review Center for Laboratory animal Experimentation Risk of Bias (SYRCLE’s RoB) tool. The SYRCLE’s RoB tool for animal experiments contains ten entries based on six types of bias: 1) Sequence generation (selection bias); 2) Baseline characteristics (selection bias); 3) Allocation concealment (selection bias); 4) Random housing (performance bias); 5) Blinding (performance bias); 6) Random outcome assessment (detection bias); 7) Blinding (detection bias); 8) Incomplete outcome data (attrition bias); 9) Selective outcome reporting (reporting bias); (10) Other sources of bias (other). The results of assessment are “yes,” “no,” and “unclear,” representing “low risk of bias,” “high risk of bias,” and “insufficient details have been reported to assess the risk of bias properly” (Hooijmans et al., 2014).

Two reviewers performed quality assessment independently, and discrepancies were discussed with a third reviewer.

### Statistical Analysis

All the outcome measures were continuous data (e.g., BG and Scr), so standardized mean difference (SMD) was considered to describe the effect sizes of the intervention effects. The confidence interval (CI) was established at 95%, and a *p* value <0.05 was considered to be statistically significant. Random-effect model was implemented to calculate the pooled results. To evaluate between-study heterogeneity, the chi-squared test and *I* ^*2*^ statistics were used. The chi-squared test with a significance level of *α* = 0.1 was used as statistical measure of heterogeneity, and *I* ^2^ > 50% was considered to represent substantial heterogeneity. Subgroup analysis was performed to investigate the possible sources of heterogeneity based on following variables if there were adequate studies: dosage (low ≤ 10 mg/kg/day; 10 < middle ≤ 20 mg/kg/day; high >20 mg/kg), intervention duration (<12 weeks; ≥ 12 weeks), DN models (type 1 DN; type 2 DN), and species (rats; mice). Sensitivity analysis was conducted to evaluate whether a single study affects the overall effect sizes by removing one study at each stage. Publication bias was assessed with the funnel plot as well as the Egger’s test (Egger et al., 1997) if there were at least 10 studies for each outcome. For Egger’s test, a *p* value less than 0.05 was considered as statistically significant (Egger et al., 1997). Meta-analysis was performed with RevMan 5.4 software.

## Results

### Study Inclusion

**FIGURE 1:**
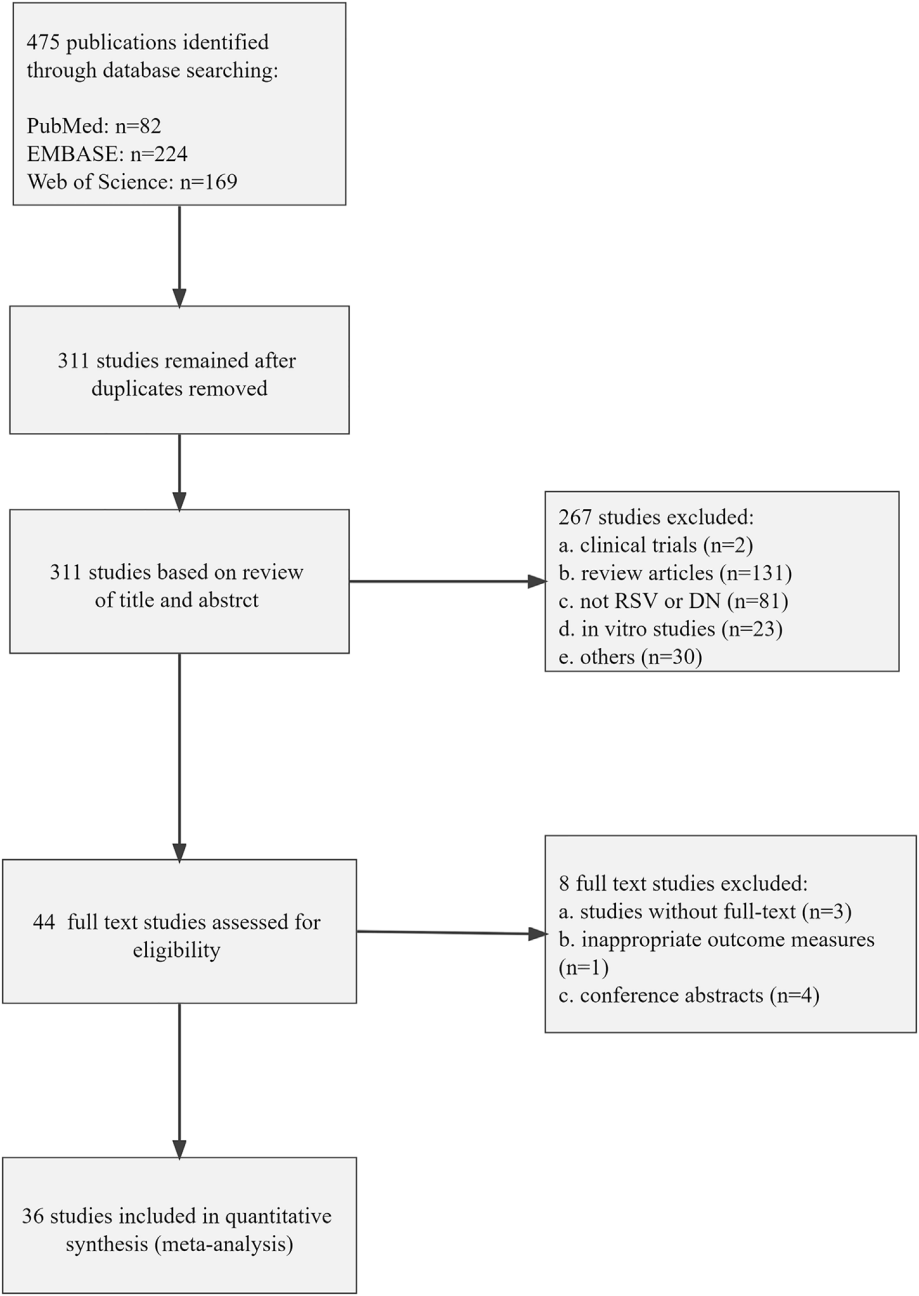
Flow diagram of the study selection process for this review.

A total number of 475 animal studies were identified through searching the databases for systematic review and meta-analysis. After removing duplicates, 311 publications remained. In screening titles and abstracts, 267 publications were excluded because of the following reasons: 1) clinical trials; 2) review articles; 3) not RSV or DN; 4) *in vitro* studies; 5) others. Then, full-text selection of the 44 remaining animal studies revealed that eight studies were ineligible due to following reasons: 1) studies without full text (*n* = 3); 2) inappropriate outcome measures (*n* = 1); 3) conference abstracts (*n* = 4). Ultimately, 36 eligible studies were included in this systematic review and meta-analysis. The process of study selection is shown in Figure 1.

### Study Characteristics

The 36 qualified studies involving 726 animals were published between 2010 and 2021. The number of all animals in the experimental group was 365 and that in the control group was 361. The animal species included mice and rats, 14 studies (38.9%) of all which used mice, and 22 studies (61.1%) used rats. The weight of rats ranged from 160 to 350 g in all studies and that of mice ranged from 16 to 30 g, and 13 studies did not report the weight of animals. There were two animal models in these studies, type 1 DN (66.7%) and type 2 DN (33.3%).

Three levels (low, medium, and high) of RSV doses were implemented in these studies and the dose ranged from 1 to 200 mg/kg/day, and two studies did not report RSV dosage. Control groups mainly included the same solvent, such as dimethyl sulfoxide (DMSO), saline, carboxymethyl cellulose (CMC), and phosphate buffer saline (PBS). Three studies (8.3%) used DMSO as the control group, ten studies (27.8%) selected saline, seven studies (19.4%) used CMC, one study (2.8%) selected phosphate buffer saline, and the remaining 15 studies (41.7%) had no treatment. The intervention duration consisted of <12 weeks and ≥12 weeks, and the duration ranged from 1 week to 32 weeks. The intervention duration were ≥12 weeks in 15 studies (41.7%) and <12 weeks in 21 studies (58.3%). The characteristics of the 36 qualified studies are outlined in Table 1. In addition, a summary table describing the RSV is shown in Supplementary Table S1.

**TABLE 1:** Characteristics of the included studies.

Study year	N= (T, C)	Species	Weight (g)	Animals models of DN	RSV dose (mg/kg/day)	Duration (weeks)	Control	Outcome index	Source of parameters
Ding et al. (2010)	6, 7	rat	NR	STZ (60 mg/kg)	10	4	1% DMSO	1. BG 2. Scr	kidney
Chang et al. (2011)	15, 7; 5, 5	rat	220–250	STZ (65 mg/kg)	1	1	saline	1. BG 2. Scr 3. BUN 4. SOD 5. TNF-α 6. IL-1β 7. IL-6	kidney
Chen et al. (2011)	7, 7	rat	NR	STZ (65 mg/kg)	2.25	8	saline	1. BG 2. Scr	kidney
Kitada et al. (2011)	18, 19; 9, 9	mice	NR	Spontaneous type 2 diabetes	NR	8	no treatment	1. BG 2. SOD	kidney
Palsamy and Subramanian, (2011)	6, 6	rat	160–180	STZ (50 mg/kg) + Nicotinamide	5	4	no treatment	1. BG 2. CAT 3. SOD 4. GPx 5.GSH 6. IL-6 7. TNF-α 8. IL-1β	kidney
Khamneh et al. (2012)	6, 6	rat	320–350	STZ (50 mg/kg) + Nicotinamide	5	16	no treatment	1. BG 2. Scr 3. CAT 4. SOD 5.GPx 6.GSH	kidney
Ramar et al. (2012)	6, 6	mice	20–25	Alloxan (75 mg/kg)	20	2	no treatment	1. BG 2. Scr 3. BUN 4. CAT 5.SOD 6.GPx 7. GSH	kidney
Wu et al. (2012)	12, 10	rat	200–240	STZ (60 mg/kg)	30	12	saline	1. BG 2. Scr 3. BUN 4. SOD 5. MDA	kidney
Huang et al. (2013)	8, 8	rat	200–220	STZ (50 mg/kg)	150	12	1% CMC	1. BG 2. Scr 3. BUN 4. SOD 5. MDA	kidney
Jiang et al. (2013)	8, 8	rat	200–220	STZ (55 mg/kg)	20	8	no treatment	1. BG 2. Scr	kidney
Kim et al. (2013)	8, 8	mice	NR	Spontaneous type 2 diabetes	20	12	0.5% CMC	1. BG 2. Scr 3. SOD	kidney
Wen et al. (2013)	10, 10	rat	180–200	STZ (50 mg/kg)	20	8	saline	1. BG	kidney
Ji et al. (2014)	10, 10	rat	200–240	STZ (60 mg/kg)	NR	12	no treatment	1. BG 2. Scr 3. BUN 4. MDA	kidney
Xu et al. (2014)	8, 8	mice	26–30	STZ (50 mg/kg)	10	12	saline	1. BG 2. Scr 3. BUN	kidney
Zheng et al. (2014)	12, 12	rat	180–220	STZ (40 mg/kg) + high-fat diet	50	32	0.5% CMC	1. BG 2. Scr 3. BUN 4. IL-1β 5. IL-6	serum
Elbe et al. (2015)	7, 7	rat	300–350	STZ (45 mg/kg)	10	4	saline	1. BG 2. Scr 3. BUN 4. SOD 5. MDA 6. CAT 7. GSH	kidney
He et al. (2016)	8, 8	mice	NR	Spontaneous type 2 diabetes	40	12	no treatment	1. BG 2. Scr	kidney
Hussein and Mahfouz, (2016)	9, 9	rat	180–220	STZ (60 mg/kg) + Nicotinamide	5	8	no treatment	1. BG 2. Scr 3. SOD 4. MDA 5. GSH 6. GPx 7. CAT	kidney
Koca et al. (2016)	9, 12; 6, 6	rat	300–350	STZ (55 mg/kg)	20	4	10% DMSO	1. BG 2. MDA 3.IL-6 4. TNF-α 5. IL-1β	kidney
Ma et al. (2016)	11, 11	rat	190–210	STZ (NR)	5	16	saline	1. BG 2. Scr 3. BUN 4. IL-1β 5. TNF-α 6. IL-6	serum
Park et al. (2016)	8, 8	mice	NR	Spontaneous type 2 diabetes	20	12	0.5% CMC	1. BG 2. Scr 3. BUN	kidney
Yan et al. (2016)	8, 8	mice	NR	Spontaneous type 2 diabetes	40	12	0.5% CMC	1. BG 2. Scr 3. BUN 4. SOD 5. MDA 6. CAT	kidney
Qiao et al. (2017)	10, 10	rat	230–270	STZ (55 mg/kg)	20	4	No treatment	1. BG 2. Scr	kidney
Xu et al. (2017)	8, 8	mice	NR	Spontaneous type 2 diabetes	100	12	0.5% CMC	1. BG 2. Scr	kidney
Al-Hussaini and Kilarkaje, (2018)	10, 10	rat	NR	STZ (55 mg/kg)	5	12	no treatment	1. BG	kidney
Bashir, (2018)	10, 10	rat	250–280	STZ (65 mg/kg)	20	8	saline	1. BG 2. Scr 3. SOD 4. MDA 5. GPx	kidney
Rehman et al. (2018)	5, 5	rat	180–250	Alloxan (120 mg/kg)	30	4	no treatment	1. BG 2. Scr 3. BUN 4.SOD 5.CAT 6.GSH	serum
Zhang et al. (2019)	6, 6	mice	20–25	STZ (140 mg/kg)	30	12	0.5% CMC	1. BG 2. BUN 3. SOD 4. MDA	kidney
Peng et al. (2019)	8, 8	rat	NR	STZ (15 mg/kg)	15	1.4	PBS	1. BG 2. Scr	kidney
Sadi et al. (2019)	9, 12	rat	NR	STZ (55 mg/kg)	20	4	10% DMSO	1. SOD 2.CAT 3.GPx	kidney
Wang et al. (2019)	9, 7	mice	16–20	Spontaneous type 1 diabetes	200	8	no treatment	1. BG	kidney
Xian et al. (2019)	10, 9	mice	20–24	Spontaneous type 1 diabetes	200	8	no treatment	1. BG 2. Scr 3. BUN	kidney
Wang et al. (2020)	13, 15; 6, 6	mice	17–23	Spontaneous type 2 diabetes	10	12	no treatment	1. BG 2. Scr 3. BUN 4. SOD 5. MDA	kidney
Xian et al. (2020)	11, 10	mice	20–24	Spontaneous type 1 diabetes	200	8	no treatment	1. BG 2. Scr 3. BUN	kidney
Zhu et al. (2020)	10, 10	rat	NR	STZ (55 mg/kg)	20	8	saline	1. Scr 2. BUN	kidney
Qi et al. (2021)	20, 20	mice	NR	STZ (35 mg/kg) + high fat-sugar diet	150	4	saline	1. BG 2. SOD 3. MDA 4. GPx	NR

Abbreviations: BG, blood glucose; BUN, blood urea nitrogen; CMC, carboxymethylcellulose; CAT, catalase; DMSO, dimethyl sulfoxide; GSH, glutathione; GPx, glutathione peroxidase; IL-1β, interleukin-1β; IL-6, interleukin-6; MDA, malondialdehyde; NR, no report; PBS, phosphate buffer saline; STZ, streptozocin; Scr, serum creatinine; SOD, superoxide dismutase; TNF-α, tumor necrosis factor-α.

Note: source of parameters: type for the oxidative/antioxidant and anti-inflammatory parameters.

### Study Quality

Random allocation to experimental and control groups was mentioned in 19 studies (52.8%), and the remaining 17 studies did not report the methods of allocation. None of the studies reported whether the distribution of relevant baseline levels was balanced between the experimental groups and control groups. None of the studies reported the blinded allocation. Random housing, blinding (performance bias), and random outcome assessment were not mentioned in all studies. All these studies had complete outcome data and reported expected outcomes. As for other sources of bias, 25 studies (69.4%) stated that there was no conflict of interest among the authors, and the remaining 11 studies (30.6%) did not mention it. The methodological quality of included studies is displayed in Table 2.

**TABLE 2:** Risk of bias of included studies.

Study year	(1)	(2)	(3)	(4)	(5)	(6)	(7)	(8)	(9)	(10)
Ding et al. (2010)	U	U	U	U	U	U	Y	Y	Y	U
Chang et al. (2011)	Y	U	U	U	U	U	Y	Y	Y	Y
Chen et al. (2011)	U	U	U	U	U	U	Y	Y	Y	U
Kitada et al. (2011)	U	U	U	U	U	U	Y	Y	Y	U
Palsamy and Subramanian, (2011)	U	U	U	U	U	U	Y	Y	Y	U
Khamneh et al. (2012)	Y	U	U	U	U	U	Y	Y	Y	Y
Ramar et al. (2012)	U	U	U	U	U	U	Y	Y	Y	U
Wu et al. (2012)	U	U	U	U	U	U	Y	Y	Y	U
Jiang et al. (2013)	Y	U	U	U	U	U	Y	Y	Y	Y
Huang et al. (2013)	Y	U	U	U	U	U	Y	Y	Y	Y
Kim et al. (2013)	U	U	U	U	U	U	Y	Y	Y	Y
Wen et al. (2013)	Y	U	U	U	U	U	Y	Y	Y	Y
Ji et al. (2014)	U	U	U	U	U	U	Y	Y	Y	U
Xu et al. (2014)	Y	U	U	U	U	U	Y	Y	Y	Y
Zheng et al. (2014)	Y	U	U	U	U	U	Y	Y	Y	U
Elbe et al. (2015)	Y	U	U	U	U	U	Y	Y	Y	Y
He et al. (2016)	Y	U	U	U	U	U	Y	Y	Y	Y
Hussein and Mahfouz, (2016)	U	U	U	U	U	U	Y	Y	Y	U
Koca et al. (2016)	Y	U	U	U	U	U	Y	Y	Y	Y
Ma et al. (2016)	U	U	U	U	U	U	Y	Y	Y	Y
Park et al. (2016)	U	U	U	U	U	U	Y	Y	Y	Y
Yan et al. (2016)	U	U	U	U	U	U	Y	Y	Y	Y
Qiao et al. (2017)	U	U	U	U	U	U	Y	Y	Y	Y
Xu et al. (2017)	Y	U	U	U	U	U	Y	Y	Y	Y
Al-Hussaini and Kilarkaje, (2018)	Y	U	U	U	U	U	Y	Y	Y	Y
Bashir, (2018)	Y	U	U	U	U	U	Y	Y	Y	Y
Rehman et al. (2018)	U	U	U	U	U	U	Y	Y	Y	Y
Zhang et al. (2019)	Y	U	U	U	U	U	Y	Y	Y	U
Peng et al. (2019)	U	U	U	U	U	U	Y	Y	Y	Y
Sadi et al. (2019)	Y	U	U	U	U	U	Y	Y	Y	Y
Wang et al. (2019)	Y	U	U	U	U	U	Y	Y	Y	Y
Xian et al. (2019)	Y	U	U	U	U	U	Y	Y	Y	Y
Wang et al. (2020)	U	U	U	U	U	U	Y	Y	Y	Y
Xian et al. (2020)	Y	U	U	U	U	U	Y	Y	Y	Y
Zhu et al. (2020)	U	U	U	U	U	U	Y	Y	Y	U
Qi et al. (2021)	Y	U	U	U	U	U	Y	Y	Y	Y

(1)Sequence gneration (2) baseline characteristics (3) allocation concealment (4) random housing (5) blinding (performance bias) (6) random outcome assessment (7) blinding (detection bias) (8) incomplete outcome data (9) selective outcome reporting (10) other sources of bias.

Y: yes; N: no; U: unclear

### Effect of Resveratrol on Blood Glucose

**FIGURE 2:**
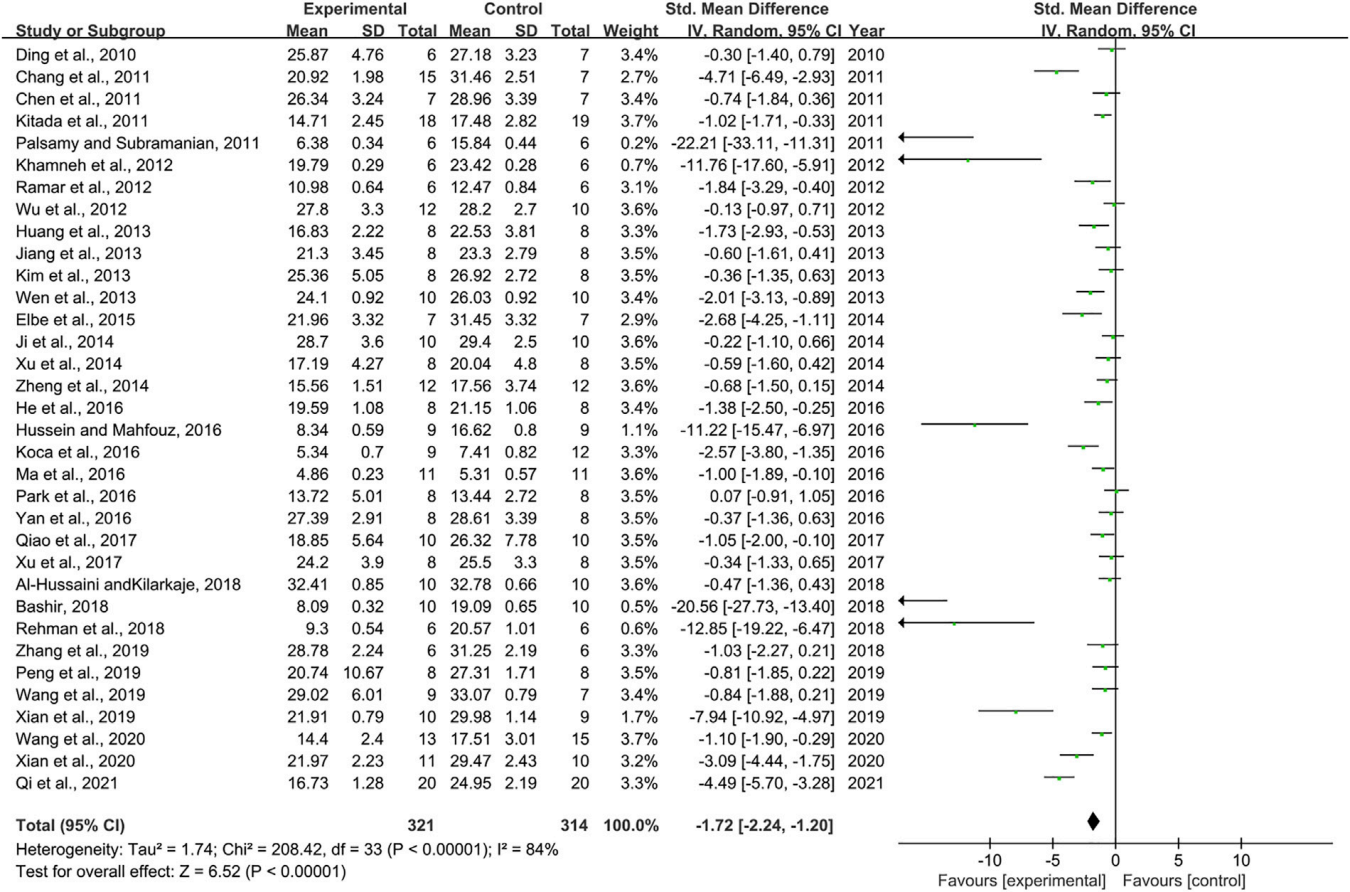
Pooled estimate of BG with RSV.

Thirty-four pair-wise comparisons reported the influence of RSV on BG. The pooled results showed that RSV could significantly decrease BG levels compared with the control group [*n* = 635, SMD = −1.72, 95% CI (−2.24, −1.20), *p* < 0.00001; Heterogeneity: X^2^ = 208.42, *p* < 0.00001; *I* ^2^ = 84%, Figure 2]. Subgroup analysis was conducted based on the DN models, intervention duration, dosage, and species. More beneficial effects were observed when studies employed type 2 DN models (*p* = 0.000), intervention duration of <12 weeks (*p* = 0.000), and rats (*p* = 0.000), as well as when they used RSV in a low-dose group (*p* = 0.000) (Supplementary Table S2). In addition, visual inspection of funnel plots showed asymmetry for the effects of RSV on BG (Supplementary Figure S1), while the result of Egger’s test was statistically significant [intercept: −5.37, 95% CI (−6.66, −4.09); *p* = 0.000].

### Effect of Resveratrol on Serum Creatinine

**FIGURE 3:**
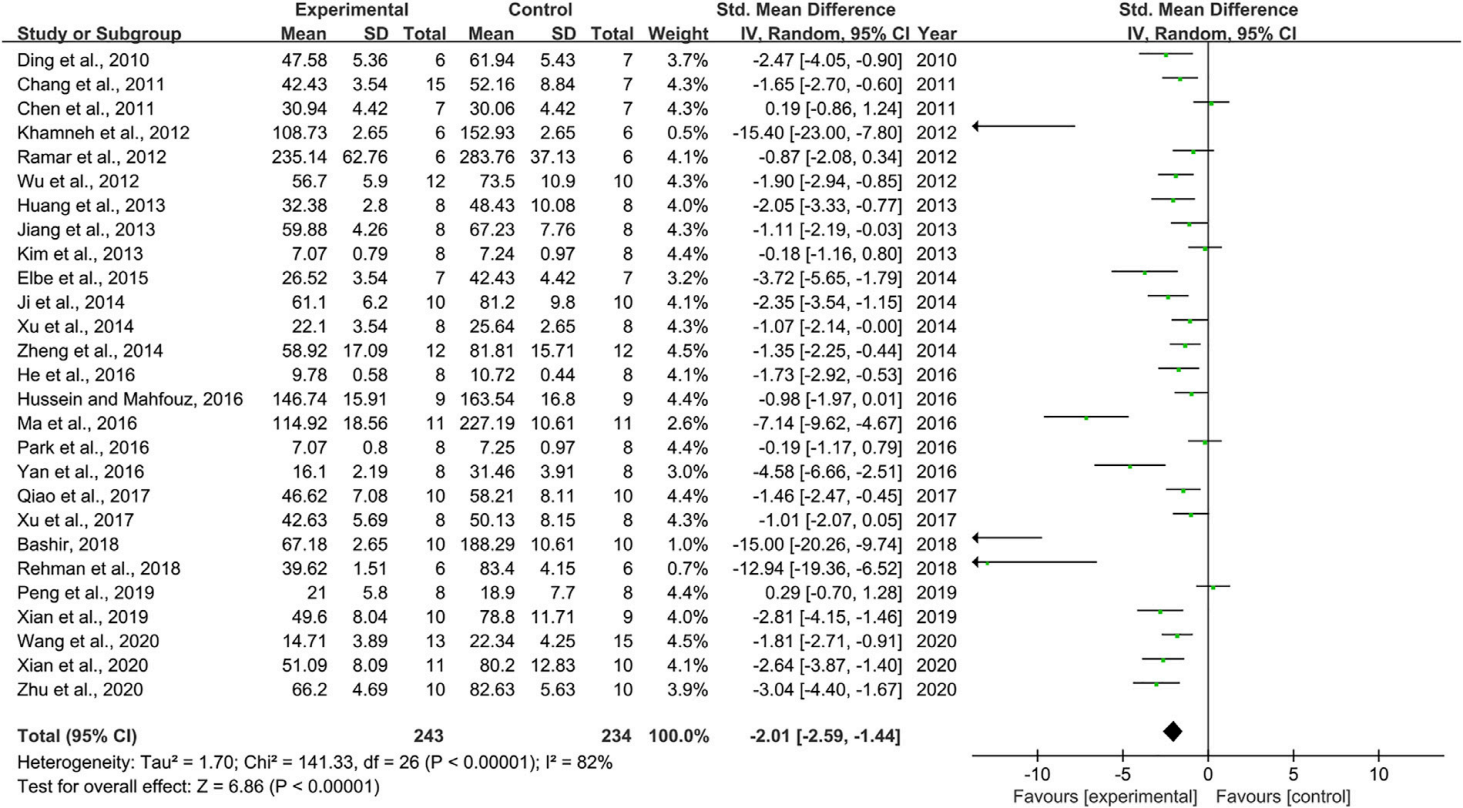
Pooled estimate of Scr with RSV.

Combining effect sizes from 27 pair-wise comparisons, a significant reduction in Scr level was observed after RSV administration, compared to that in the control group [*n* = 477, SMD = −2.01, 95% CI (−2.59, −1.44), *p* < 0.00001; Heterogeneity: X^2^ = 141.33, *p* < 0.00001; *I* ^2^ = 82%, Figure 3]. Subgroup analysis was performed according to DN models, intervention duration, dosage, and species. More beneficial effects were observed when studies applied type 1 DN models (*p* = 0.000), rats (*p* = 0.000), and intervention duration of <12 weeks (*p* = 0.000), as well as when they used RSV in a low-dose group (*p* = 0.000) (Supplementary Table S2). Furthermore, visual inspection of funnel plots showed asymmetry for the effects of RSV on Scr (Supplementary Figure S2), and the result of Egger’s test was statistically significant [intercept: −5.63, 95% CI (−7.15, −4.10); *p* = 0.000].

### Effect of Resveratrol on Blood Urea Nitrogen

**FIGURE 4:**
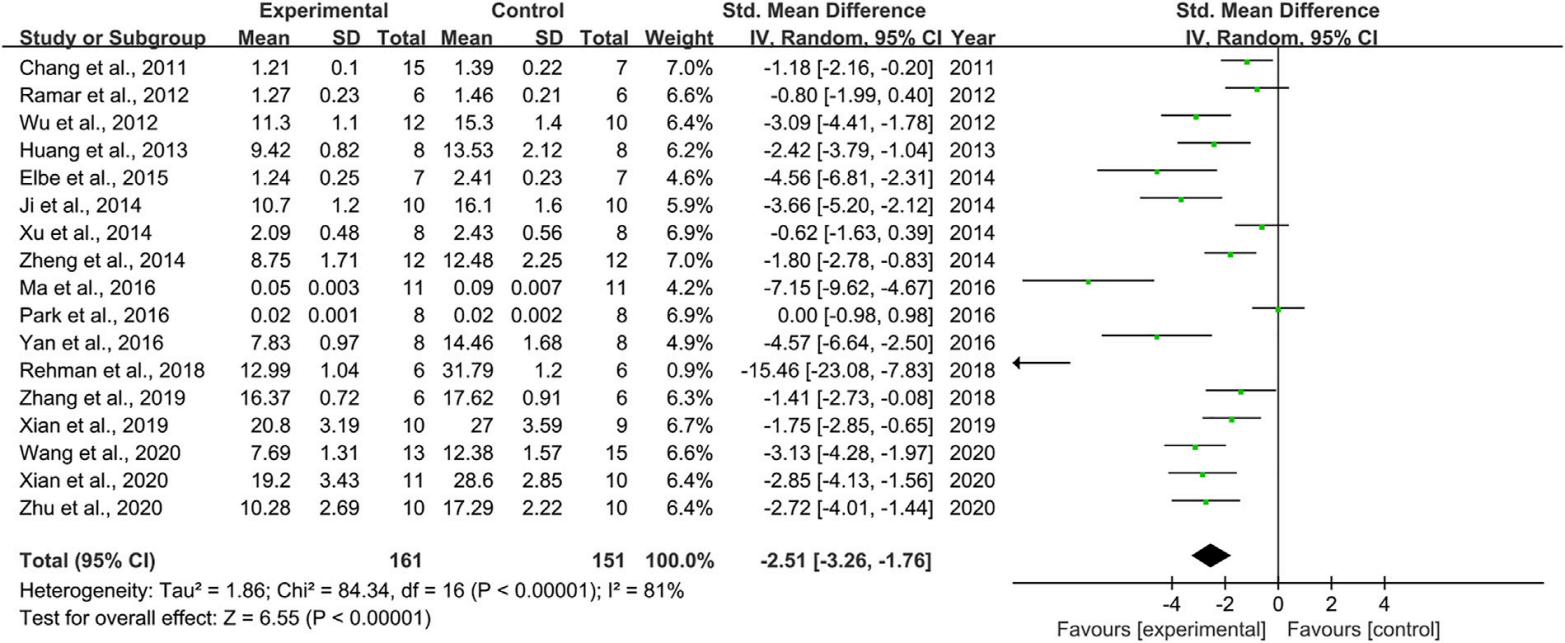
Pooled estimate of BUN with RSV.

Seventeen pair-wise comparisons reported the influence of RSV on BUN. The pooled results suggested that RSV could significantly decrease BUN level compared with the control group [*n* = 312, SMD = −2.51, 95% CI (−3.26, −1.76), *p* < 0.00001; Heterogeneity: X^2^ = 84.34, *p* < 0.00001; *I* ^2^ = 81%, Figure 4]. The included studies were stratified according to variables including DN models, intervention duration, dosage, and species. Better therapeutic effects were observed when studies used type 1 DN models (*p* = 0.000), intervention duration of ≥12 weeks (*p* = 0.000), and rats (*p* = 0.000), as well as when they used RSV in a low-dose group (*p* = 0.000) (Supplementary Table S2). Funnel plots showed asymmetry for the effects of RSV on BUN (Supplementary Figure S3), while the result of Egger’s test was statistically significant [intercept: −5.84, 95% CI (−8.09, −3.59); *p* = 0.019].

### Effect of Resveratrol on Superoxide Dismutase

**FIGURE 5:**
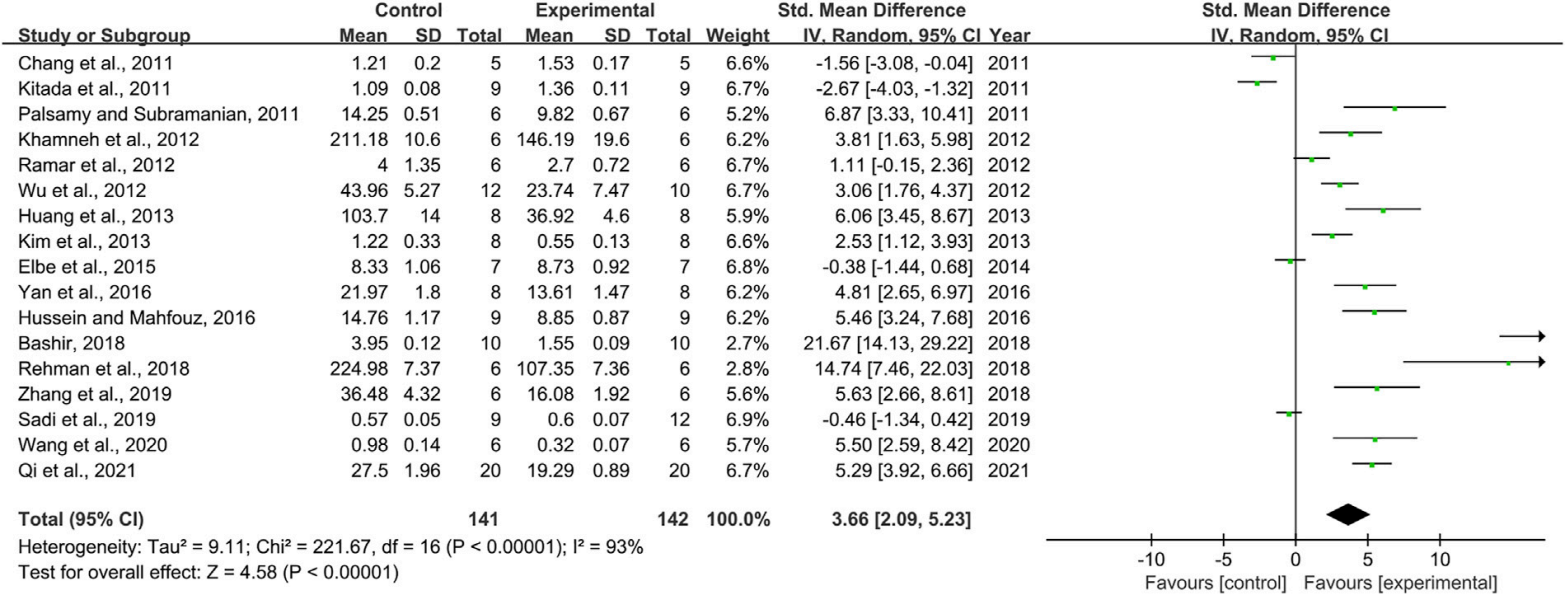
Pooled estimate of SOD with RSV.

Seventeen pair-wise comparisons mentioned the influence of RSV on SOD. The pooled effect sizes indicated that RSV could significantly increase the level of SOD compared with a control group [*n* = 283, SMD = 3.66, 95% CI (2.09, 5.23), *p* < 0.00001; Heterogeneity: X^2^ = 221.67, *p <* 0.00001; *I* ^2^ = 93%, Figure 5]. Subgroup analysis was conducted based on DN models, intervention duration, dosage, and species. More beneficial effects were demonstrated when studies used type 2 DN models (*p* = 0.002), intervention duration of ≥12 weeks (*p =* 0.000), and rats (*p* = 0.000), as well as studies that employed high dosage (*p* = 0.000) (Supplementary Table S2). Funnel plots showed asymmetry for the effects of RSV on SOD (Supplementary Figure S4), while the result of Egger’s test was statistically significant [intercept: 6.01, 95% CI (2.95, 9.08); *p* = 0.001].

### Effect of Resveratrol on Malondialdehyde

**FIGURE 6:**
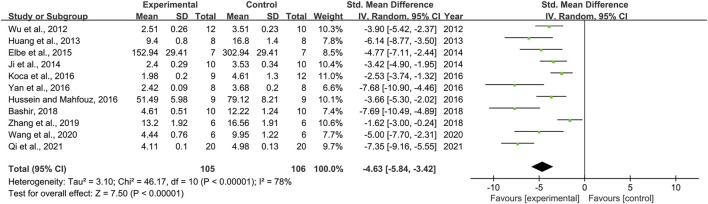
Pooled estimate of MDA with RSV.

Effect sizes for MDA were pooled from a total of 11 pair-wise comparisons. There was a significant association of RSV with MDA level [*n* = 211, SMD = −4.63, 95% CI (−5.84, −3.42), *p* < 0.00001; Heterogeneity: X^2^ = 46.17, *p* < 0.00001; *I* ^2^ = 78%, Figure 6]. The included studies were stratified according to variables including DN models, intervention duration, dosage, and species. More beneficial effects were found when studies used type 2 DN models (*p* = 0.000), intervention duration of <12 weeks (*p* = 0.000), and mice (*p* = 0.002), as well as when they used RSV in a high-dose group (*p* = 0.000) (Supplementary Table S2). Furthermore, funnel plots indicated asymmetry for the effects of RSV on MDA (Supplementary Figure S5), and the result of Egger’s test was statistically significant [intercept: −5.34, 95% CI (−8.63, −2.05); *p* = 0.005].

### Effect of Resveratrol on Catalase

**FIGURE 7:**
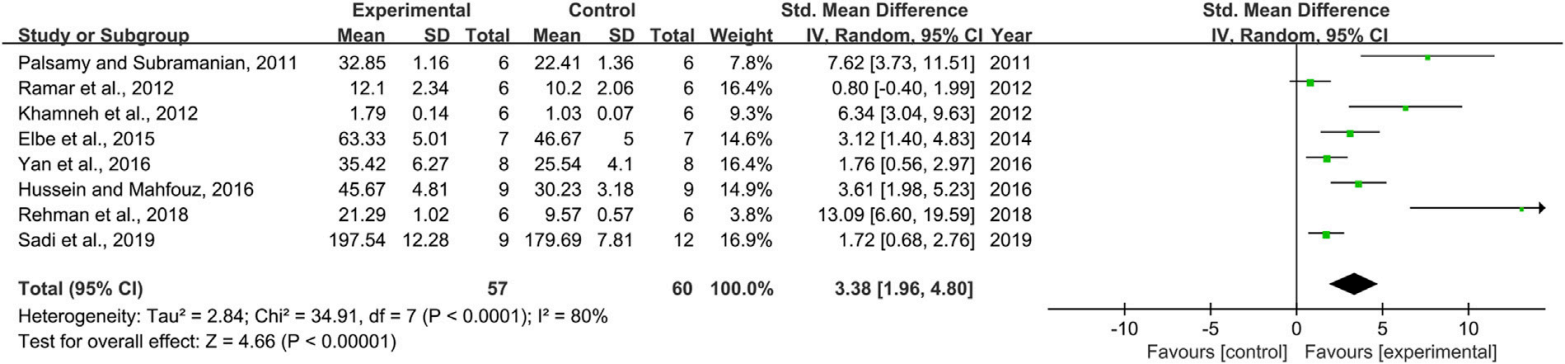
Pooled estimate of CAT with RSV.

Effect sizes for CAT were pooled from a total of eight pair-wise comparisons. There was a significant association of RSV with CAT level [*n* = 117, SMD = 3.38, 95% CI (1.96, 4.80), *p* < 0.00001; Heterogeneity: X^2^ = 34.91, *p* < 0.0001; *I* ^2^ = 80%, Figure 7]. Subgroup analysis was performed according to DN models, intervention duration, dosage, and species. More beneficial effects were observed when studies applied type 2 DN models (*p* = 0.000), intervention duration of <12 weeks (*p* = 0.000), and rats (*p* = 0.000), as well as studies that employed low dosage (*p* = 0.000) (Supplementary Table S2). However, publication bias was not conducted on CAT as less than 10 studies were included.

### Effect of Resveratrol on Glutathione

**FIGURE 8:**
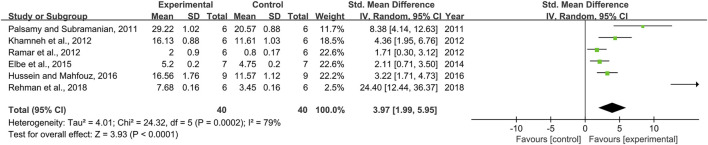
Pooled estimate of GSH with RSV.

Six pair-wise comparisons mentioned the impact of RSV on GSH. The pooled effect sizes showed that RSV could significantly increase the GSH level compared with the control group [*n* = 80, SMD = 3.97, 95% CI (1.99, 5.95), *p* < 0.0001; Heterogeneity: X^2^ = 24.32, *p =* 0.0002; *I* ^2^ = 79%, Figure 8]. Subgroup analysis was performed according to DN models, intervention duration, dosage, and species. More beneficial effects were observed when studies applied type 2 DN models (*p* = 0.000), intervention duration of ≥12 weeks (*p* = 0.000), and rats (*p* = 0.000), as well as when they used RSV in a low-dose group (*p* = 0.000) (Supplementary Table S2). Nevertheless, publication bias was not conducted on GSH as less than 10 studies were included.

### Effect of Resveratrol on Glutathione Peroxidase

**FIGURE 9:**
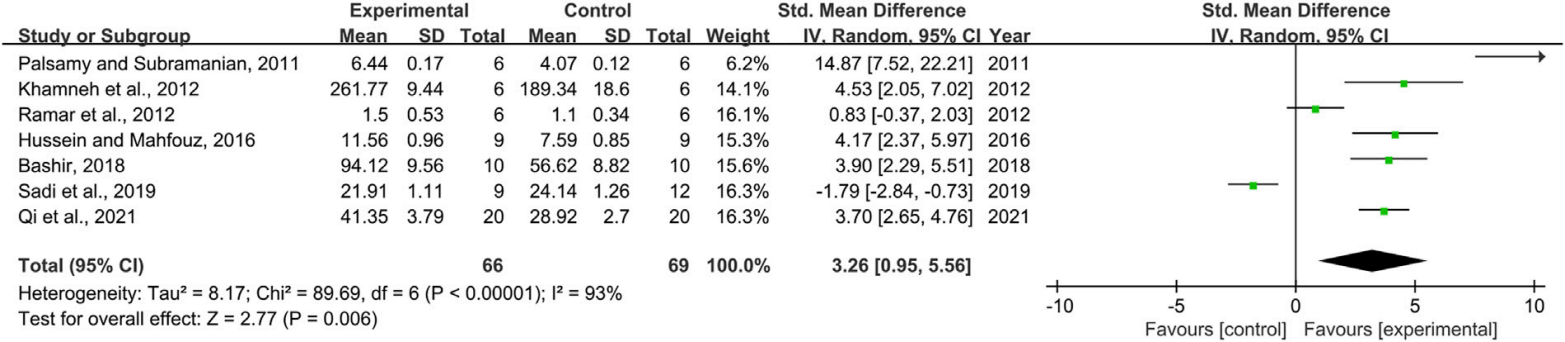
Pooled estimate of GPx with RSV.

Seven pair-wise comparisons reported the impact of RSV on GPx. The pooled effect sizes showed that RSV could significantly increase the GPx level compared with control group [*n* = 135, SMD = 3.26, 95% CI (0.95, 5.56), *p* = 0.006; Heterogeneity: X^2^ = 89.69, *p <* 0.00001; *I* ^2^ = 93%, Figure 9]. The included studies were stratified according to variables including DN models, intervention duration, dosage, and species. Better therapeutic effects were observed when studies used type 2 DN models (*p* = 0.000), intervention duration of ≥12 weeks (*p* = 0.000), and rats (*p* = 0.028), as well as when they used RSV in a low-dose group (*p* = 0.001) (Supplementary Table S2). However, publication bias was not conducted on GPx as less than 10 studies were included.

### Effect of Resveratrol on TNF-α

**FIGURE 10:**
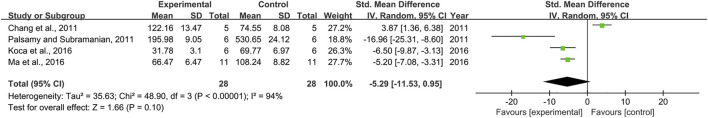
Pooled estimate of TNF-α with RSV.

Combining effect sizes from four pair-wise comparisons, no significant decrease in the level of TNF-α was observed after RSV administration, compared to that in the control group [*n* = 56, SMD = −5.29, 95% CI (−11.53, 0.95), *p* = 0.1; Heterogeneity: X^2^ = 48.90, *p* < 0.00001; *I* ^2^ = 94%, Figure 10]. Because meta-analysis indicated that there was no significant association of RSV with the level of TNF-α, subgroup analysis was not performed on TNF-α. In addition, publication bias was not conducted on TNF-α as less than 10 studies were included.

### Effect of Resveratrol on IL-6

**FIGURE 11:**
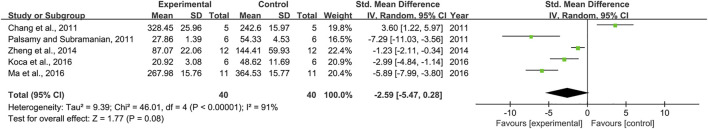
Pooled estimate of IL-6 with RSV.

As for the effect on IL-6, 5 pair-wise comparisons mentioned the influence of RSV on this outcome. The pooled effect sizes showed that RSV did not significantly decrease IL-6 level compared with the control group [*n* = 80, SMD = −2.59, 95% CI (−5.47, 0.28), *p* = 0.08; Heterogeneity: X^2^ = 46.01, *p <* 0.00001; *I* ^2^ = 91%, Figure 11]. Because meta-analysis indicated that there was no significant association of RSV with the level of IL-6, subgroup analysis was not performed on IL-6. Furthermore, publication bias was not conducted on IL-6 as less than 10 studies were included.

### Effect of Resveratrol on IL-1β

**FIGURE 12:**
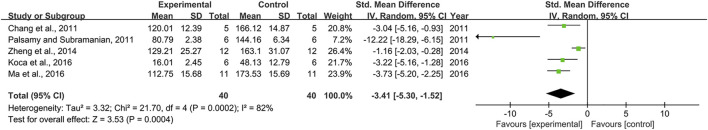
Pooled estimate of IL-1β with RSV.

Effect sizes for IL-1β were pooled from a total of 5 pair-wise comparisons. There was a significant association of RSV with IL-1β level (*n* = 80, SMD = −3.41, 95% CI (−5.30, −1.52), *p* = 0.0004; Heterogeneity: X^2^ = 21.70, *p* = 0.0002; *I* ^2^ = 82%, Figure 12]. Subgroup analysis was conducted according to DN models, intervention duration, and dosage. More beneficial effects were observed when studies employed type 1 DN models (*p* = 0.000), intervention duration of <12 weeks (*p* = 0.004), as well as when they used RSV in a low-dose group (*p* = 0.001) (Supplementary Table S2). Subgroup analysis was not conducted according to species because all included studies applied rat models of DN. Publication bias was not performed on IL-6 as less than 10 studies were included.

### Sensitivity Analysis

For BG, Scr, BUN, SOD, MDA, CAT, GSH, GPx, and IL-1β, the sensitivity analysis was conducted by removing one study at each stage, and the results indicated that no individual study significantly affected the pooled effect sizes.

Nevertheless, TNF-α and IL-6 were influenced in one study (Chang et al., 2011), and there was a significant association of RSV with TNF-α and IL-6 levels after removing this study [Before sensitivity analysis: TNF-α: SMD = −5.29, 95% CI (−11.53, 0.95), *p* = 0.1; IL-6: SMD = -2.59, 95% CI (−5.47, 0.28), *p* = 0.08. After sensitivity analysis: TNF-α: SMD = −7.64, 95% CI (−11.80, −3.48), *p* = 0.0003; IL-6: SMD = −4.03, 95% CI (−6.67, −1.38), *p* = 0.003].

## Discussion

**FIGURE 13:**
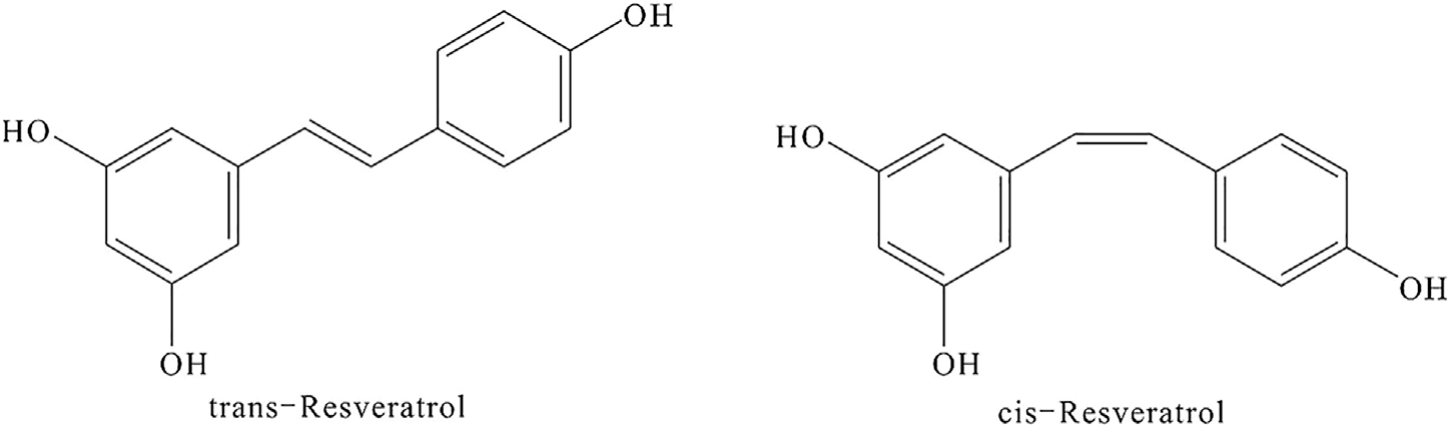
The chemical structure of RSV.

RSV is a phytoalexin phenolic metabolite produced in response to environmental stress (Gowd et al., 2020). Its chemical name is 3,4′,5-trihydroxy-trans-stilbene or 5-[(1E)-2-(4-hydroxyphenyl)ethenyl]-1,3-benzenediol. RSV has a CAS registry number of 501-36-0, a molecular weight of 228.24, and an empirical formula of C_14_H_12_O_3_. The appearance of RSV is that of an off-white powder with a melting point of 254–257°C. It is soluble in water at 25°C and easily soluble in organic solvents such as ethyl acetate, ether, chloroform, methanol, ethanol, acetone, and ethyl acetate. RSV exists in two isoforms *cis-* and *trans-*resveratrol, and its chemical structure is shown in Figure 13.

The present systematic review and meta-analysis mainly intended to evaluate the anti- oxidant and anti-inflammatory properties of RSV when used in the treatment of DN. The results showed that RSV was significantly associated with a lower level of MDA and higher levels of SOD, CAT, GSH, and GPx. With regard to pro-inflammatory cytokines, RSV can significantly reduce the IL-1β level, but it did not effectively decrease the levels of TNF-α and IL-6. Current evidence supports the antioxidant and anti-inflammatory properties of RSV, but its relationship with some inflammatory mediators such as IL-6 and TNF-α in animals with diabetic nephropathy needs further elucidation.

Hyperglycemia is a predominant cause underlying the development and progression of DN (DeFronzo et al., 2021), and improving BG levels plays an important role in renal protection. However, whether RSV can decrease BG levels remains controversial. Recent studies indicated that RSV could reduce BG levels in animal models of DN (Xian et al., 2020; Qi et al., 2021). Other studies suggested that RSV had no significant hypoglycemic effect (Park et al., 2016; Yan et al., 2016). In this meta-analysis, compared to the control group, the level of BG was significantly decreased in RSV-treated groups. In addition, DN models might influence the hypoglycemic effect of RSV according to subgroup analysis. The efficacy of RSV on reducing BG was more significant in type 2 DN animal models, but the hypoglycemic effect of RSV in type 1 DN animal models was relatively diminished. Type 1 DN most commonly results from an autoimmune disorder in which pancreatic beta cells are destroyed, while type 2 DN is characterized by an insufficient response to circulatory insulin by peripheral tissues because of insulin resistance (Li et al., 2021). Hence, the insulin resistance is the primary difference between type 1 DN and type 2 DN. In a double-blind clinical trial, the insulin resistance and the level of insulin in type 2 diabetic patients were significantly improved by RSV treatment (Zare Javid et al., 2017). Another study also showed that RSV treatment can improve insulin sensitivity in type 2 diabetic patients (Brasnyó et al., 2011). Thus, the ability of RSV to improve insulin resistance may be the reason it has a more powerful hypoglycemic effect in type 2 DN.

Scr and BUN are the most important markers that reflect renal function status. Early reports found that RSV treatment could significantly reduce the levels of Scr and BUN in animal models of DN. However, previous studies also indicated that there was no significant difference in Scr between treatment and control groups (Peng et al., 2019). With regard to BUN, one study showed that RSV was not associated with a lower level of BUN (Xu et al., 2014). Therefore, conflicting actions of RSV in animal models of DN still exist in the current researches. Our meta-analysis suggested that RSV treatment was significantly associated with lower levels of Scr and BUN. This result indicated that RSV has a beneficial effect on DN animal models.

Dose-response effects and time-response effects play an important role in clinical medication. However, no animal studies reported the dose-response effects and time-response effects of RSV when used in the treatment of DN. In the meta-analysis reported here, with regard to BG, Scr, and BUN, the greatest effects were recorded in low-dose groups rather than in high-dose groups. Moreover, in a randomized, double-blind, placebo-controlled clinical trial, high-dose RSV had no significant influence on BG and insulin sensitivity in obese human subjects (Poulsen et al., 2013). According to these findings, variability in dosage of RSV may influence the efficacy, and low-dose RSV may be more suitable for the treatment of DN. Thus, it is necessary to notice that the dosage of RSV should be increased slowly starting from a low dosage in clinical use. Nevertheless, whether an excessive dose of RSV will suppress its therapeutic effects in the treatment of DN should be further investigated. For time-response effects, subgroup analysis indicated that intervention duration of RSV can influence the hypoglycemic effect, and more beneficial effects were observed when studies had a drug administration time of <12 weeks. However, there was no significant difference in the influence of RSV on Scr and BUN between intervention duration <12 weeks and intervention duration >12 weeks. Therefore, it is difficult to determine the appropriate intervention duration based on current evidence. According to the above findings, attention should be paid to the following two aspects in the future studies: first, both patients with type 1 DN and type 2 DN need to be included in clinical trials. During clinical trials, the levels of insulin and BG must be strictly monitored in order to determine which type of DN is more sensitive to RSV treatment. Second, the particular multi-arm clinical trials (two or more experimental intervention groups with a common control group) need to be conducted. In multi-arm clinical trials, multiple levels of dosage or intervention duration are required to evaluate the dose-response effects or time-response effects and determine the optimal dosage or intervention duration.

Oxidative stress is considered to be an etiologic factor in the pathogenesis of DN, and an imbalance between excess generation of pro-oxidants and antioxidants including SOD, CAT, GPx, and GSH is believed to play an important role in modulating kidney tissue damage such as renal tubular cell death, tubulointerstitial fibrosis, and glomerular mesangial expansion (Singh et al., 2011). SOD is a major antioxidant enzyme found in mitochondria and cytoplasm, and can change super-oxide into molecular oxygen and hydrogen peroxide. GPx utilizes GSH to detoxify hydrogen peroxide to lipid peroxides and water, along with serving as a peroxynitrite reductase (Yamaguchi et al., 1998). The increased generation of CAT in the proximal tubules of transgenic diabetic animals can mitigate tubular apoptosis and interstitial fibrosis (Brezniceanu et al., 2008). MDA is an unsaturated aldehyde produced by the oxidation of polyunsaturated fatty acids (Mandlik et al., 2021). SOD, CAT, GPx, GSH, and MDA are used *in vivo* as markers of oxidative stress. Previous studies found that RSV treatment could effectively mitigate renal dysfunction by recovering SOD, CAT, GPx, and GSH activity (Khamneh et al., 2012; Ramar et al., 2012). Consistent with the previous findings, in our meta-analysis the increased levels of SOD, CAT, GPx, and GSH were observed following RSV treatment. Recent work suggested that the MDA content in control group is significantly higher than that in RSV-treated group (Zhang et al., 2019; Wang et al., 2020). This result was also supported by our study. Interestingly enough, our subgroup analysis indicated that DN models may affect the antioxidant effects, the increased levels of SOD, CAT, GPx, and GSH and decreased MDA content were all observed when studies used models for type 2 DN. One point that should be considered in relation to this result is the better hypoglycemic effect of RSV in type 2 DN. Chronic hyperglycemia plays a major part in the occurrence of oxidative stress and impairment of the antioxidant response (Nishikawa et al., 2000), so the better hypoglycemic effect in type 2 DN might contribute to a higher antioxidant capacity of RSV in type 2 DN. These findings raise a question regarding the potential value of RSV as a promising candidate in the treatment of type 2 DN instead of type 1 DN. With regard to dose-response effects, the increased levels of SOD and GSH and decreased MDA level were recorded in high-dose groups. However, a higher level of CAT and GPx was observed in studies with low-dose groups. As for time-response effects, the increased SOD, GSH, and GPx levels were found when studies had intervention duration ≥12 weeks, but the decreased MDA content and enhanced CAT levels were observed in intervention duration <12 weeks. These contradictory results might be due to the small sample size in subgroup analysis. For example, only one study of GSH and GPx was included in the high-dose and intervention duration ≥12 weeks groups. Thus, the dose-response effects and time-response effects of RSV in antioxidant capacity in the treatment of DN should be further clarified through increasing sample size.

Pro-inflammatory cytokines including TNF-α, IL-1β, and IL-6 are critical mediators in the development and progression of DN. In animal models of DN, the renal levels of TNF-α, IL-1β, and IL-6 are significantly upregulated when compared with those in healthy animals (Koca et al., 2016; Ma et al., 2016). TNF-α is cytotoxic to glomerular mesangial and epithelial cells, and can lead directly to kidney tissue damage (Palsamy and Subramanian, 2011). The IL-1β level is related to increased permeability of vascular endothelial cells (Pérez-Morales et al., 2019). IL-6 can induce fibronectin expression, promote mesangial cell proliferation, and increase the permeability of endothelial cells (Navarro-González and Mora-Fernández, 2008). A previous study indicated that RSV obviously decreased the level of IL-1β but enhanced that of IL-6 and TNF-α in the DN animal models (Chang et al., 2011). Other studies suggested that RSV was significantly associated with a lower level of TNF-α, IL-1β, and IL-6 (Koca et al., 2016; Ma et al., 2016). Based on the above results, there is no consensus about the anti-inflammatory property of RSV in the treatment of DN. Our meta-analysis found that RSV did not decrease the levels of IL-6 and TNF-α but was significantly associated with a low level of IL-1β. Therefore, the anti-inflammatory effects of RSV still need to be confirmed in more studies.

The molecular mechanisms of antioxidant and anti-inflammatory properties of RSV in the treatment of DN may be related to multiple signaling pathways. Recent studies demonstrated that the mechanisms of RSV against oxidative stress were mainly through activating the silent information regulator T1 (SIRT1) signaling pathway to inhibit the production of reactive oxygen species (ROS), reduce the expression of the receptor for advanced glycation end products (RAGE), activate adenosine monophosphate-activated protein kinase (AMPK), inhibit the activation of nuclear factor kappa B (NF-κB), regulate NF-E2-related-factor 2-Kelch-like ECH associated protein 1 (Nrf2-Keap1) signaling pathway, and decrease the expression of glutathione S-transferase (Deng et al., 2018). In addition, one study found that RSV can upregulate the expression of 3-hydroxy-3-methylglutaryl coenzyme in the db/db mice kidney, which promotes the degradation of insulin-like growth factor 1 receptor protein (IGF-1R), thereby enhancing the activity of antioxidant enzymes (Yan et al., 2016). Forkhead box O3a (FoxO3a) plays a critical role in the regulation of oxidative stress. Recent studies indicated that RSV treatment can increase SIRT1 deacetylase activity, subsequently decreasing the expression of acetylated-FoxO3a and inhibiting the oxidative stress caused by hyperglycemia both *in vitro* and *in vivo* (Wang et al., 2017). Furthermore, several studies confirmed that Forkhead box O1 (FoxO1) is also associated with oxidative stress. With an increase in MDA levels and a decrease in SOD activity, the expression of FoxO1 was reduced. Nevertheless, FoxO1 expression was increased and SIRT1 deacetylase activity was enhanced after RSV treatment. These results suggest that RSV can reduce renal oxidative stress injury by regulating SIRT1/FoxO1 signaling pathway (Deng et al., 2018). Moreover, in the pathological state of hyperglycemia and hemodynamic disorder, a large number of renal macrophages are infiltrated, and multiple inflammatory cytokines such as IL-1β, TNF-α, and IL-6 are released. Among them, the activation of the NF-κB signal transduction pathway is considered to be an important intermediate link for many signal pathways. Several studies suggested that RSV can significantly reduce the expression of IL-6, IL-1β, intercellular cell adhesion molecule-1 (ICAM-1), and other inflammatory cytokines in the kidney tissue through modulating AMPK/NF-κB, Akt/NF-κB, phosphoinositide 3-kinases/protein kinase B (PI3K/Akt) signaling pathway, and the pancreatic insulin signal transduction pathway (Chang et al., 2011; Ojima et al., 2013; Xu et al., 2014; Sadi et al., 2015). In addition, RSV could inhibit the expression of inflammatory cytokines such as TNF-α and IL-6 by inhibiting the phosphorylation of extracellular signal-regulated kinase 1/2 (Chen and Wei, 2015).

Angiogenesis is the formation of new blood vessels from the pre-existing vascular system (Zent and Pozzi, 2007). The process of angiogenesis mainly consists of four events including detachment from basement membranes, proliferation and migration of endothelial cells, formation of endothelial tubes, and maturation of new vessels (Pandya et al., 2006). Under physiological conditions, these processes are regulated by pro-angiogenic and anti-angiogenic factors. However, increased expression of pro-angiogenic factors and decreased expression of anti-angiogenic factors within the glomerulus were observed during the occurrence and progression of DN. This leads to increased proliferation and migration of endothelial cells, resulting in the formation of immature and leaky vessels (Zent and Pozzi, 2007). One study reported that increased density in glomerular capillaries resulting from glomerular neovascularization and an increasing number of efferent arterioles at the glomerular vascular pole were seen in biopsy specimens of patients with type 1 diabetes and animal models of DN (Osterby et al., 1999; Guo et al., 2005). Among the pro-angiogenic factors, vascular endothelial growth factor (VEGF) is probably the most effective upregulated permeability factor in DN. VEGF is produced primarily by podocytes in the glomerulus, and high glucose can increase the synthesis of VEGF in cultured podocytes and tubules (Iglesias-de la Cruz et al., 2002; Kim et al., 2005). At the early stage of DN, VEGF was found to be higher than in normal mice, and VEGF mRNA was also significantly upregulated, and was positively correlated with the urinary microprotein excretion rate. Moreover, one previous study observed that several pathological changes similar to those of DN, such as proteinuria, glomerular injury, mesangial hyperplasia, and basal membrane thickening in mice with overexpression of VEGF, could be reversed when drug-induced overexpression of VEGF was inhibited (Jiang and Ding, 2017). It has been confirmed that administration of VEGF antibodies can ameliorate renal injury in diabetic mice (Flyvbjerg et al., 2002). Furthermore, a study of renal biopsy specimens from diabetic patients showed increased expression of VEGF and increased VEGF-receptor activation in mildly impaired glomerulus. This was accompanied by increased endothelial cell proliferation, suggesting that VEGF activation in mildly affected diabetic kidney might lead to increased glomerular angiogenesis (Hohenstein et al., 2006). Angiopoietins (Angs) are a family of vascular growth factors that regulate vascular remodeling, maturation, and stability. The Angs family includes Ang 1, Ang 2, Ang 3, and Ang 4, and they interact with tyrosine kinase receptors (Tie 1 and Tie 2) (Tao et al., 2021). Ang 1 and Ang 2 have opposite effects on endothelial cell function. Ang 1 can stabilize the adhesion of endothelial cells and promote the maturation of new capillaries by binding to the Tie 2 receptor. Ang 2 blocks Ang 1/Tie 2 signaling, which leads to increased angiogenesis, vascular instability, and subsequent leakage. In the kidneys, Ang 1 counteracts the effects of VEGF and increases the trans-endothelial resistance of cultured glomerular endothelial cells. In contrast, upregulation of Ang 2 was found in the mouse model of type 1 DN and mesangial cells exposed to high glucose levels (Yamamoto et al., 2004; Ichinose et al., 2005). According to the above findings, altering the activity of these mediators such as VEGF, Ang 1, and Ang 2 might be beneficial in preventing the progression of DN. One study showed that RSV blocked diabetes-induced increase of VEGF expression (Kim et al., 2012). However, another study did not find significant changes in the level of VEGF mRNA associated with vascular remodeling after RSV treatment (Yar et al., 2012). Therefore, results on anti-angiogenic effects of RSV are contradictory (Toro et al., 2019; Pietras-Baczewska et al., 2021). Recent work found that RSV could decrease the expression of VEGF and Ang 2, and enhance the expression of Tie 2 in the rat model of DN. However, no change in Ang 1 expression was detected (Wen et al., 2013). Based on these results, whether RSV can achieve anti-angiogenic effects by regulating anti-angiogenic factors and pro-angiogenic factors remains to be further studied.

Every medical intervention comes with the risk, great or small, of adverse effects. All clinicians should comprehensively consider the adverse aspects of interventions. A study of the immunotoxicity of RSV in male B_6_C_3_F_1_/N mice found that after 28 days of RSV treatment (0, 156, 312, 625, 1,250, and 2,500 mg/kg/day), the humoral, cell-mediated, and innate immune function were not changed (Huang et al., 2020). However, some studies reported that RSV treatment could result in DNA damage and reduce several DNA repair pathways, which can activate cytotoxic and apoptotic pathways (Shaito et al., 2020). A human trial indicated that RSV at a dosage of 1,000 mg/day or above could suppress cytochrome P450 isoenzymes including CYP2D6, CYP3A4, and CYP2C9 (Detampel et al., 2012). It has been reported that 450 mg/day of RSV is a safe dose for a 60-kg person (Wahab et al., 2017). In this meta-analysis, no study reported the occurrence of adverse effects. The reasons for this could be that the dosage and intervention duration of RSV were within a reasonable range, which may not be sufficient to produce adverse effects. Moreover, researchers did not report any adverse effects that occurred in the experiment. On the basis of the above findings, it is necessary to pay attention to the following two points in future research: first, the association between dose and intervention duration of RSV and adverse effects needs to be further studied. Second, some researchers did not report adverse effects, which would make people mistakenly believe that there were no adverse effects from this intervention. Hence, researchers should report in detail whether there are adverse effects in the experiments.

Several limitations should be considered in this systematic review and meta-analysis. First, a number of studies did not report baseline characteristics between experimental groups and control groups. Second, Egger’s test and asymmetry of funnel plots showed that publication bias existed, which could exaggerate the therapeutic effects of RSV. Therefore, the positive findings on RSV should be interpreted with caution. Third, some studies did not report a randomization process.

## Conclusion

In this meta-analysis, RSV can exert its antioxidant effect by reducing the levels of MDA and restoring the activities of SOD, CAT, GSH, and GPx. With regard to pro-inflammatory cytokines, RSV had a positive effect on the reduction of IL-1β. However, the analysis indicated that RSV had no effect on IL-6 and TNF-α levels, probably because of the methodological quality of the studies and their heterogeneity. Current evidence supports the antioxidant and anti-inflammatory properties of RSV, but its relationship with the levels of some inflammatory mediators such as IL-6 and TNF-α in animals with DN needs further elucidation.

## Data Availability

The original contributions presented in the study are included in the article/Supplementary Material, further inquiries can be directed to the corresponding author.
