# Supplementary material for: Antioxidant and Anti-inflammatory Properties of Resveratrol in Diabetic Nephropathy: A Systematic Review and Meta-analysis of Animal Studies
Source: Front Pharmacol. 2022 Mar 9;13:841818. doi: 10.3389/fphar.2022.841818 (PMC8959544; doi:10.3389/fphar.2022.841818)
Supplement: Supplementary file 1 [file Table1.docx]

**Table S1** A summary table describing the RSV.

| **Study year** | **Compound,**  **concentration** | **Source** | **Purity** | **Quality control reported?** |
| --- | --- | --- | --- | --- |
| Ding et al., 2010 | Pure compound | Sigma-Aldrich, St. Louis, MO, USA | ≥ 99% | Y- HPLC |
| Chang et al., 2011 | Pure compound | Sigma-Aldrich, St. Louis, MO, USA | ≥ 99% | Y- HPLC |
| Chen et al., 2011 | Pure compound | Sigma-Aldrich, St. Louis, MO, USA | ≥ 99% | Y- HPLC |
| Kitada et al., 2011 | Pure compound | Sigma-Aldrich, St. Louis, MO, USA | ≥ 99% | Y- HPLC |
| Palsamy and Subramanian, 2011 | Pure compound | Sigma-Aldrich, St. Louis, MO, USA | ≥ 99% | Y- HPLC |
| Khamneh et al., 2012 | Pure compound | Cayman chem., Ann Arbor, MI, USA | NR | NR |
| Ramar et al., 2012 | Pure compound | Sigma-Aldrich, St. Louis, MO, USA | ≥ 99% | Y- HPLC |
| Wu et al., 2012 | NR | NR | NR | NR |
| Jiang et al., 2013 | Pure compound | JF-NATURAL ,Tianjin, China | >98% | NR |
| Huang et al., 2013 | Pure compound | Zelang, Nanjing, China | >98% | Y- HPLC |
| Kim et al., 2013 | Pure compound | Sigma-Aldrich, St. Louis, MO, USA | ≥ 99% | Y- HPLC |
| Wen et al., 2013 | Pure compound | Copalyton Chemical Materials Co., Ltd, Shanghai, China | NR | NR |
| Ji et al., 2014 | NR | NR | NR | NR |
| Xu et al., 2014 | NR | NR | NR | NR |
| Zheng et al., 2014 | NR | NR | NR | NR |
| Elbe et al., 2015 | Pure compound | Molekula, UK | NR | NR |
| He et al., 2016 | Pure compound | Sigma-Aldrich, St. Louis, MO, USA | ≥ 99% | Y- HPLC |
| Hussein and Mahfouz, 2016 | Pure compound | Sigma-Aldrich, St. Louis, MO, USA | ≥ 99% | Y- HPLC |
| Koca et al., 2016 | Pure compound | Molekula , Gill ingham, Dorset, UK | NR | NR |
| Ma et al., 2016 | Pure compound | Cayman chem., Ann Arbor, MI, USA | NR | NR |
| Park et al., 2016 | Pure compound | Sigma-Aldrich, St. Louis, MO, USA | ≥ 99% | Y- HPLC |
| Yan et al., 2016 | Pure compound | Sigma-Aldrich, St. Louis, MO, USA | ≥ 99% | Y- HPLC |
| Qiao et al., 2017 | Pure compound | Cayman chem., Ann Arbor, MI, USA | NR | NR |
| Xu et al., 2017 | Pure compound | Sigma-Aldrich, St. Louis, MO, USA | ≥ 99% | Y- HPLC |
| Al-Hussaini and Kilarkaje, 2018 | NR | NR | NR | NR |
| Bashir, 2018 | Pure compound | Sigma-Aldrich, St. Louis, MO, USA | ≥99% | Y-HPLC |
| Rehman et al., 2018 | Pure compound | Bristol Mayer Biotech Pakistan | NR | NR |
| Zhang et al., 2019 | Pure compound | Sigma-Aldrich, St. Louis, MO, USA | ≥99% | Y-HPLC |
| Peng et al., 2019 | Pure compound | Sigma-Aldrich, St. Louis, MO, USA | ≥99% | Y-HPLC |
| Sadi et al., 2019 | NR | NR | NR | NR |
| Wang et al., 2019 | Pure compound | Sigma-Aldrich; Merck KGaA | ≥99% | Y-HPLC |
| Xian et al., 2019 | NR | NR | NR | NR |
| Wang et al., 2020 | Pure compound | Sigma-Aldrich, St. Louis, MO, USA | ≥99% | Y-HPLC |
| Xian et al., 2020 | Pure compound | Sigma-Aldrich; Merck KGaA | ≥99% | Y-HPLC |
| Zhu et al., 2020 | Pure compound | Sigma-Aldrich, St. Louis, MO, USA | ≥99% | Y-HPLC |
| Qi et al., 2021 | NR | NR | NR | NR |

**Abbreviations:** (HPLC: high performance liquid chromatography; NR: no report).
