# Supplementary material for: Antioxidant and Anti-inflammatory Properties of Resveratrol in Diabetic Nephropathy: A Systematic Review and Meta-analysis of Animal Studies
Source: Front Pharmacol. 2022 Mar 9;13:841818. doi: 10.3389/fphar.2022.841818 (PMC8959544; doi:10.3389/fphar.2022.841818)
Supplement: Supplementary file 2 [file Table2.docx]

**Table S2**. Subgroup analysis for each outcome measures.

| **Variables** | **No. of Trials** | **SMD [95%CI]** | ***P* value** | ***I^2^*(%)** | ***P*-heterogeneity** | |
| --- | --- | --- | --- | --- | --- | --- |
| **blood glucose** |  |  |  |  |  | |
| DN models |  |  |  |  |  | |
| type 1 DN | 22 | -1.70 [-2.31, -1.09] | 0.000 | 82 | 0.000 | |
| type 2 DN | 12 | -1.84 [-2.84, -0.85] | 0.000 | 88 | 0.000 | |
| dosage |  |  |  |  | |  |
| low | 11 | -2.33 [-3.47, -1.19] | 0.000 | 87 | | 0.000 |
| medium | 9 | -1.43 [-2.39, -0.47] | 0.003 | 83 | | 0.000 |
| high | 12 | -1.93 [-2.89, -0.97] | 0.000 | 87 | | 0.000 |
| NR | 2 | -1.67 [-1.45, 0.12] | 0.096 | 50 | | 0.157 |
| duration |  |  |  |  | |  |
| <12 weeks | 19 | -2.97 [-3.91, -2.03] | 0.000 | 88 | | 0.000 |
| ≥12 weeks | 15 | -0.68 [-1.03, -0.32] | 0.000 | 47 | | 0.023 |
| species |  |  |  |  | |  |
| rats | 20 | -2.07 [-2.86, -1.29] | 0.000 | 86 | | 0.000 |
| mice | 14 | -1.44 [-2.13, -0.76] | 0.000 | 82 | | 0.000 |
| **Scr** |  |  |  |  | |  |
| DN models |  |  |  |  | |  |
| type 1 DN | 18 | -2.32 [-3.09, -1.54] | 0.000 | 83 | | 0.000 |
| type 2 DN | 9 | -1.47 [-2.29, -0.65] | 0.000 | 77 | | 0.000 |
| dosage |  |  |  |  | |  |
| low | 9 | -2.33 [-3.49, -1.18] | 0.000 | 84 | | 0.000 |
| medium | 8 | -1.40 [-2.51, -0.29] | 0.014 | 85 | | 0.000 |
| high | 9 | -2.26 [-3.04, -1.47] | 0.000 | 68 | | 0.001 |
| NR | 1 | -2.35 [-3.54, -1.15] | 0.000 | － | | － |
| duration |  |  |  |  | |  |
| <12 weeks | 14 | -2.10 [-3.00, -1.21] | 0.000 | 84 | | 0.000 |
| ≥12 weeks | 13 | -1.97 [-2.73, -1.20] | 0.000 | 80 | | 0.000 |
| species |  |  |  |  | |  |
| rats | 17 | -2.48 [-3.35, -1.61] | 0.000 | 86 | | 0.000 |
| mice | 10 | -1.53 [-2.21, -0.85] | 0.000 | 71 | | 0.000 |
| **BUN** |  |  |  |  | |  |
| DN models |  |  |  |  | |  |
| type 1 DN | 13 | -2.62 [-3.49, -1.75] | 0.000 | 80 | | 0.000 |
| type 2 DN | 4 | -2.23 [-3.95, -0.51] | 0.011 | 88 | | 0.000 |
| dosage |  |  |  |  |  | |
| low | 5 | -3.05 [-4.85, -1.24] | 0.001 | 89 | 0.000 | |
| medium | 3 | -1.13 [-2.69, 0.42] | 0.154 | 82 | 0.004 | |
| high | 8 | -2.61 [-3.51, -1.71] | 0.000 | 68 | 0.003 | |
| NR | 1 | -3.66 [-5.20, -2.12] | 0.000 | － | － | |
| duration |  |  |  |  |  | |
| <12 weeks | 7 | -2.44 [-3.59, -1.28] | 0.000 | 77 | 0.000 | |
| ≥12 weeks | 10 | -2.57 [-3.61, -1.52] | 0.000 | 84 | 0.000 | |
| species |  |  |  |  |  | |
| rats | 9 | -3.32 [-4.46, -2.19] | 0.000 | 80 | 0.000 | |
| mice | 8 | -1.76 [-2.70, -0.82] | 0.000 | 79 | 0.000 | |
| **SOD** |  |  |  |  |  | |
| DN models |  |  |  |  |  | |
| type 1 DN | 9 | 3.33 [1.30, 5.35] | 0.001 | 92 | 0.000 | |
| type 2 DN | 8 | 3.83 [1.37, 6.28] | 0.002 | 93 | 0.000 | |
| dosage |  |  |  |  |  | |
| low | 6 | 3.06 [0.33, 5.79] | 0.028 | 92 | 0.000 | |
| medium | 4 | 3.12 [0.21, 6.03] | 0.035 | 93 | 0.000 | |
| high | 6 | 5.23 [3.64, 6.81] | 0.000 | 68 | 0.009 | |
| NR | 1 | -2.67 [-4.03, -1.32] | 0.000 | － | － | |
| duration |  |  |  |  |  | |
| <12 weeks | 10 | 3.20 [1.02, 5.38] | 0.004 | 94 | 0.000 | |
| ≥12 weeks | 7 | 4.08 [3.04, 5.13] | 0.000 | 45 | 0.093 | |
| species |  |  |  |  |  | |
| rats | 10 | 4.23 [2.05, 6.41] | 0.000 | 93 | 0.000 | |
| mice | 7 | 3.05 [0.60, 5.49] | 0.015 | 93 | 0.000 | |
| **MDA** |  |  |  |  |  | |
| DN models |  |  |  |  |  | |
| type 1 DN | 7 | -3.96 [-5.26, -2.66] | 0.000 | 74 | 0.001 | |
| type 2 DN | 4 | -5.80 [-7.90, -3.69] | 0.000 | 72 | 0.013 | |
| dosage |  |  |  |  |  | |
| low | 3 | -4.22 [-5.43, -3.02] | 0.000 | 0 | 0.611 | |
| medium | 2 | -4.95 [-9.99, 0.10] | 0.054 | 91 | 0.001 | |
| high | 5 | -5.17 [-7.60, -2.74] | 0.000 | 87 | 0.000 | |
| NR | 1 | -3.42 [-4.90, -1.95] | 0.000 | － | － | |
| duration |  |  |  |  |  | |
| <12 weeks | 5 | -5.06 [-7.13, -2.98] | 0.000 | 84 | 0.000 | |
| ≥12 weeks | 6 | -4.26 [-5.82, -2.71] | 0.000 | 74 | 0.002 | |
| species |  |  |  |  |  | |
| rats | 7 | -4.21 [-5.31, -3.11] | 0.000 | 61 | 0.016 | |
| mice | 4 | -5.30 [-8.63, -1.97] | 0.002 | 90 | 0.000 | |
| **CAT** |  |  |  |  |  | |
| DN models |  |  |  |  |  | |
| type 1 DN | 4 | 2.66 [0.68, 4.64] | 0.008 | 82 | 0.001 | |
| type 2 DN | 4 | 4.29 [1.97, 6.61] | 0.000 | 79 | 0.003 | |
| dosage |  |  |  |  |  | |
| low | 4 | 4.51 [2.76, 6.25] | 0.000 | 54 | 0.089 | |
| medium | 2 | 1.31 [0.40, 2.21] | 0.005 | 24 | 0.251 | |
| high | 2 | 6.96 [-4.11, 18.03] | 0.218 | 91 | 0.001 | |
| duration |  |  |  |  |  | |
| <12 weeks | 6 | 3.48 [1.70, 5.25] | 0.000 | 82 | 0.000 | |
| ≥12 weeks | 2 | 3.78 [-0.67, 8.24] | 0.096 | 85 | 0.011 | |
| species |  |  |  |  |  | |
| rats | 6 | 4.65 [2.63, 6.67] | 0.000 | 80 | 0.000 | |
| mice | 2 | 1.28 [0.33, 2.22] | 0.008 | 20 | 0.264 | |
| **GSH** |  |  |  |  |  | |
| DN models |  |  |  |  |  | |
| type 1 DN | 3 | 3.45 [0.08, 6.81] | 0.045 | 85 | 0.001 | |
| type 2 DN | 3 | 4.63 [2.30, 6.96] | 0.000 | 62 | 0.075 | |
| dosage |  |  |  |  |  | |
| low | 4 | 3.76 [1.98, 5.54] | 0.000 | 67 | 0.030 | |
| medium | 1 | 1.71 [0.30, 3.12] | 0.017 | － | － | |
| high | 1 | 24.40 [12.44, 36.37] | 0.000 | － | － | |
| duration |  |  |  |  |  | |
| <12 weeks | 5 | 3.99 [1.67, 6.30] | 0.001 | 82 | 0.000 | |
| ≥12 weeks | 1 | 4.36 [1.95, 6.76] | 0.000 | － | － | |
| species |  |  |  |  |  | |
| rats | 5 | 4.89 [2.32, 7.4] | 0.000 | 81 | 0.000 | |
| mice | 1 | 1.71 [0.30, 3.12] | 0.017 | － | － | |
| **GPx** |  |  |  |  |  | |
| DN models |  |  |  |  |  | |
| type 1 DN | 3 | 0.93 [-2.13, 3.99] | 0.550 | 94 | 0.000 | |
| type 2 DN | 4 | 4.70 [2.81, 6.59] | 0.000 | 66 | 0.031 | |
| dosage |  |  |  |  |  | |
| low | 3 | 5.97 [2.50, 9.43] | 0.001 | 74 | 0.021 | |
| medium | 3 | 0.93 [-2.13, 3.99] | 0.550 | 94 | 0.000 | |
| high | 1 | 3.70 [2.65, 4.76] | 0.000 | － | － | |
| duration |  |  |  |  |  | |
| <12 weeks | 6 | 3.06 [0.55, 5.58] | 0.017 | 94 | 0.000 | |
| ≥12 weeks | 1 | 4.53 [2.05, 7.02] | 0.000 | － | － | |
| species |  |  |  |  |  | |
| rats | 5 | 4.12 [0.44, 7.81] | 0.028 | 95 | 0.000 | |
| mice | 2 | 2.28 [-0.53, 5.10] | 0.112 | 92 | 0.000 | |
| **IL-1β** |  |  |  |  |  | |
| DN models |  |  |  |  |  | |
| type 1 DN | 3 | -3.42 [-4.45, -2.40] | 0.000 | 0 | 0.848 | |
| type 2 DN | 2 | -6.26 [-17.07, 4.55] | 0.256 | 92 | 0.000 | |
| dosage |  |  |  |  |  | |
| low | 3 | -4.81 [-7.74, -1.87] | 0.001 | 75 | 0.019 | |
| medium | 1 | -3.22 [-5.16, -1.28] | 0.010 | － | － | |
| high | 1 | -1.16 [-2.03, -0.28] | 0.001 | － | － | |
| duration |  |  |  |  |  | |
| <12 weeks | 3 | -4.78 [-8.04, -1.52] | 0.004 | 76 | 0.017 | |
| ≥12 weeks | 2 | -2.37 [-4.89, 0.15] | 0.065 | 88 | 0.003 | |
