# Supplementary material for: Antioxidant and Anti-inflammatory Properties of Resveratrol in Diabetic Nephropathy: A Systematic Review and Meta-analysis of Animal Studies
Source: Front Pharmacol. 2022 Mar 9;13:841818. doi: 10.3389/fphar.2022.841818 (PMC8959544; doi:10.3389/fphar.2022.841818)
Supplement: Supplementary file 3 [file DataSheet1.PDF]

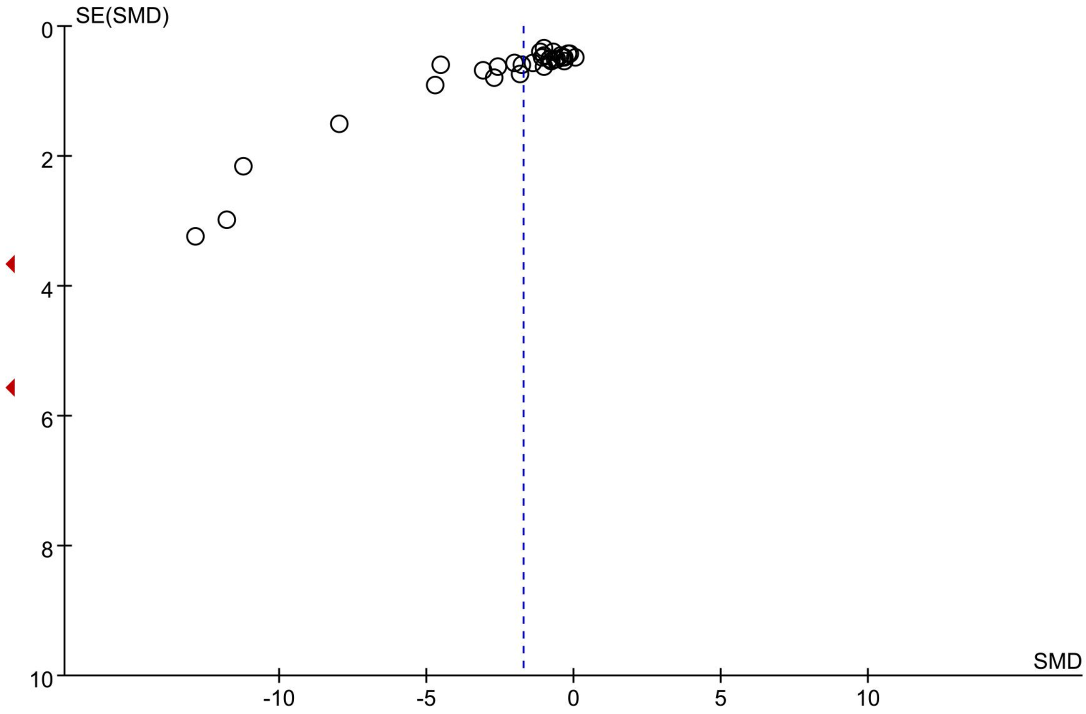

Figure S1 Funnel plot for efficacy of RSV on BG.

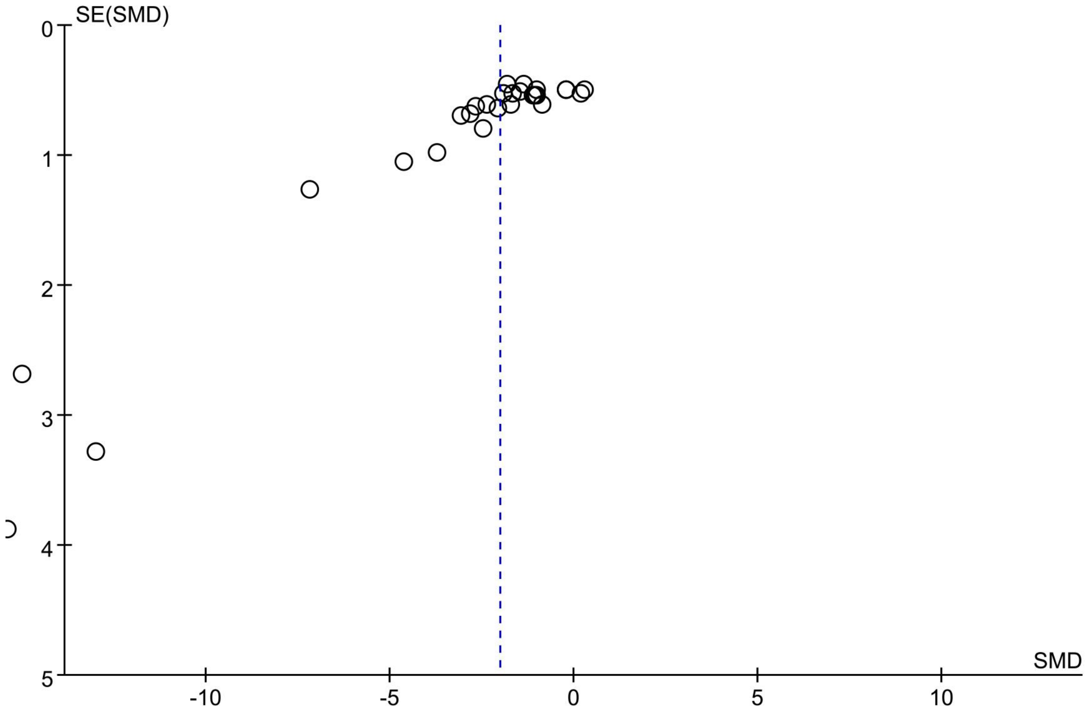

Figure S2 Funnel plot for efficacy of RSV on Scr.

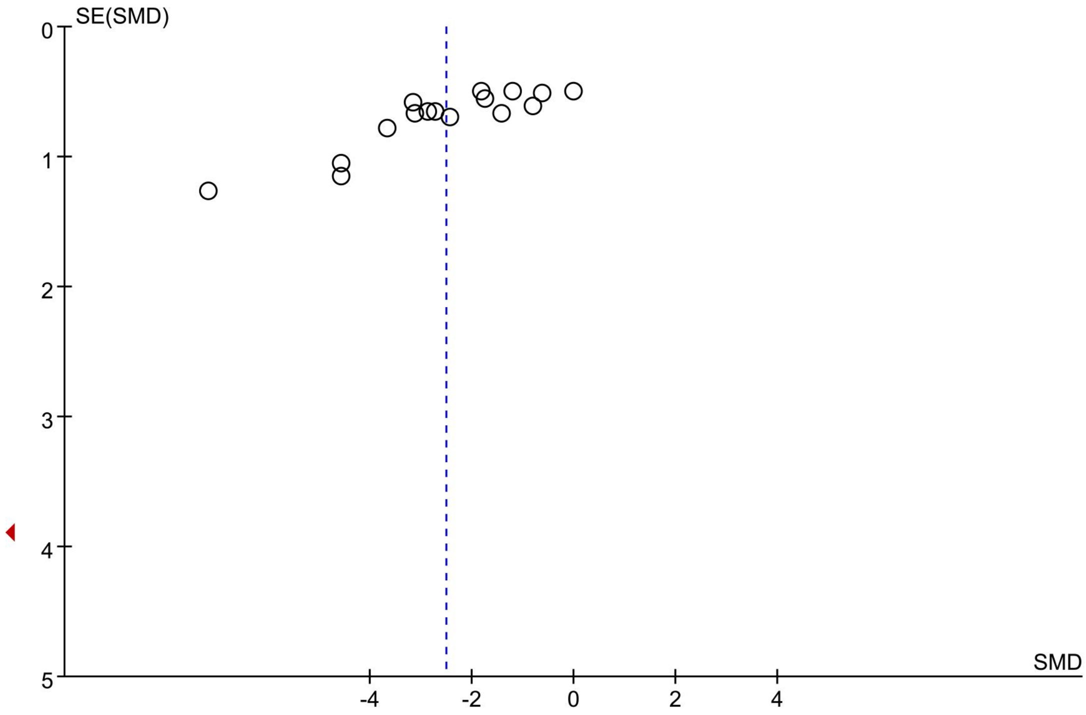

Figure S3 Funnel plot for efficacy of RSV on BUN.

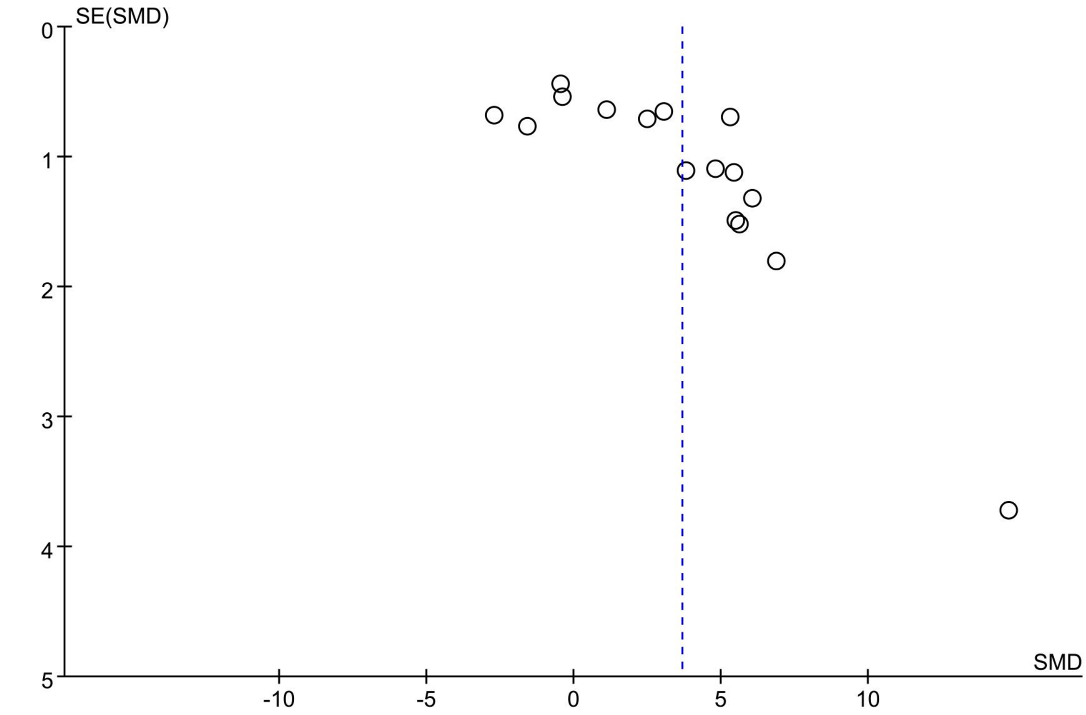

Figure S4 Funnel plot for efficacy of RSV on SOD.

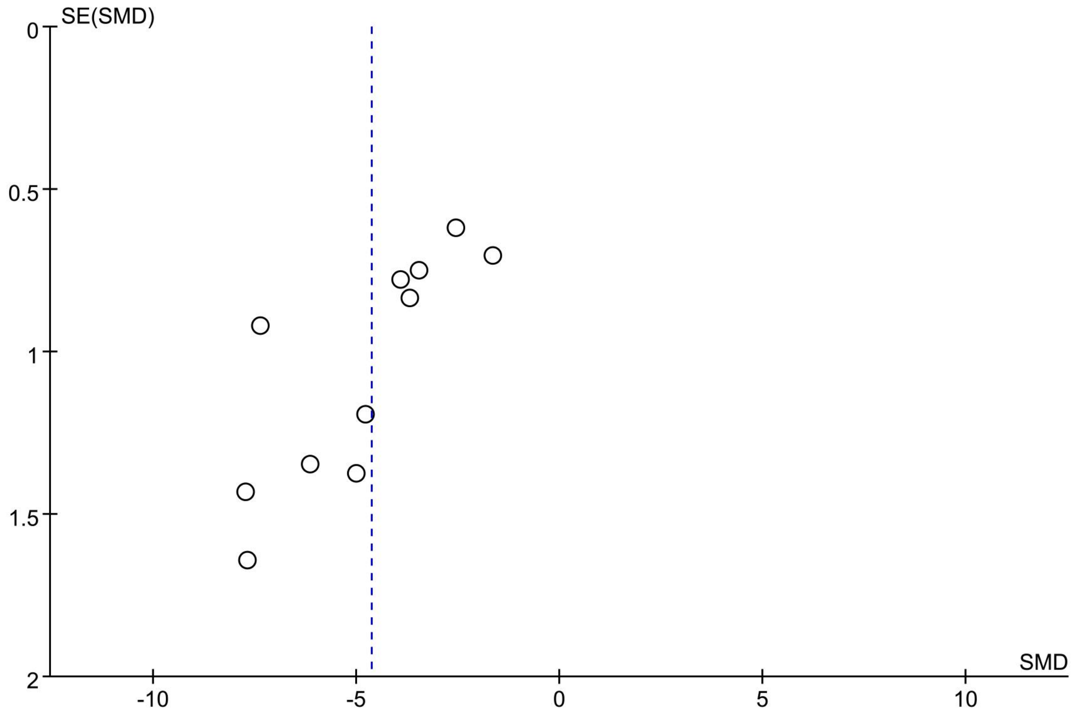

Figure S5 Funnel plot for efficacy of RSV on MDA.
